# Compound Sarcopenia in Hospitalized Patients with Cirrhosis Worsens Outcomes with Increasing Age

**DOI:** 10.3390/nu13020659

**Published:** 2021-02-18

**Authors:** Nicole Welch, Amy Attaway, Annette Bellar, Hayder Alkhafaji, Adil Vural, Srinivasan Dasarathy

**Affiliations:** 1 Department of Gastroenterology and Hepatology, Cleveland Clinic, Cleveland, OH 44195, USA; welchn@ccf.org; 2 Department of Inflammation and Immunity, Cleveland Clinic, Cleveland, OH 44195, USA; bellara@ccf.org (A.B.); h.alkhafaji@hotmail.com (H.A.); vurala@ccf.org (A.V.); 3 Department of Pulmonary Medicine, Cleveland Clinic, Cleveland, OH 44195, USA; attawaa@ccf.org

**Keywords:** sarcopenia, cirrhosis, aging, clinical outcomes, cost of stay, inpatient mortality

## Abstract

Background: There are limited data on outcomes of older patients with chronic diseases. Skeletal muscle loss of aging (primary sarcopenia) has been extensively studied but the impact of secondary sarcopenia of chronic disease is not as well evaluated. Older patients with chronic diseases have both primary and secondary sarcopenia that we term compound sarcopenia. We evaluated the clinical impact of compound sarcopenia in hospitalized patients with cirrhosis given the increasing number of patients and high prevalence of sarcopenia in these patients. Design: The Nationwide Inpatients Sample (NIS) database (years 2010–2014) was analyzed to study older patients with cirrhosis. Since there is no universal hospital diagnosis code for “muscle loss”, we used a comprehensive array of codes for “muscle loss phenotype” in the international classification of diseases-9 (ICD-9). A randomly selected 2% sample of hospitalized general medical population (GMP) and inpatients with cirrhosis were stratified into 3 age groups based on age-related changes in muscle mass. In-hospital mortality, length of stay (LoS), cost of hospitalization (CoH), comorbidities and discharge disposition were analyzed. Results. Of 517,605 hospitalizations for GMP and 106,835 hospitalizations for treatment of cirrhosis or a cirrhosis-related complication, 207,266 (40.4%) GMP and 29,018 (27.7%) patients with cirrhosis were >65 years old, respectively. Muscle loss phenotype in both GMP and inpatients with cirrhosis 51–65 years old and >65 years old was significantly (*p* < 0.001 for all) associated with higher mortality, LoS, and CoH compared to those ≤50 years old. Patients >65 years old with cirrhosis and muscle loss phenotype had higher mortality (adjusted OR: 1.06, 95% CI [1.04, 1.08] and CoH (adjusted odds ratio (OR): 1.10, 95% confidence interval (CI) [1.04, 1.08])) when compared to >65 years old GMP with muscle loss phenotype. Muscle loss in younger patients with cirrhosis (≤50 years old) was associated with worse outcomes compared to GMP >65 years old. Non-home discharges (nursing, skilled, long-term care) were more frequent with increasing age to a greater extent in patients with cirrhosis with muscle loss phenotype for each age stratum. Conclusion: Muscle loss is more frequent in older patients with cirrhosis than younger patients with cirrhosis and older GMP. Younger patients with cirrhosis had clinical outcomes similar to those of older GMP, suggesting an accelerated senescence in cirrhosis. Compound sarcopenia in older patients with cirrhosis is associated with higher inpatient mortality, increased LoS, and CoH compared to GMP with sarcopenia.

## 1. Introduction

With the increasing age of the population in the United States [1], the number of older patients with cirrhosis continues to rise [2]. Older patients use significantly greater health care resources than younger patients, especially during hospitalization [3,4,5]. In the setting of increasing longevity in different societies, the proportion of hospitalized older adults with cirrhosis, compared to those who are younger, continues to increase [6,7,8]. Aging adversely affects hepatic function, responses to injury, and complications of cirrhosis [9,10,11]. Loss of skeletal muscle mass, or sarcopenia, is one of the most frequent complications in cirrhosis that contributes to mortality, morbidity, decreased quality of life (QoL), and poor post-liver transplant (LT) outcomes [12,13,14,15,16]. The term sarcopenia was initially used to describe the syndrome of reduced muscle mass with impaired contractile function in older adults [17]; however, with widespread clinical recognition of muscle loss in chronic diseases, aging-related muscle loss is considered to be “primary sarcopenia,” while “secondary sarcopenia” is that which occurs in chronic diseases [17]. In older patients with chronic diseases, primary (age-related) sarcopenia and secondary (disease-related) sarcopenia are likely to be additive, but have not been evaluated. We, therefore, refer to the presence of sarcopenia in older patients with chronic disease as “compound sarcopenia.” Hospitalized patients are themselves, a priori, at risk for sarcopenia due to underlying disease and frequent comorbidities [18,19]. Hospitalization aggravates muscle loss due to a combination of factors including immobilization, dietary alterations, and prolonged fasting and has been referred to as “acute sarcopenia” [18,20]. Acute sarcopenia of hospitalization is likely additive to compound sarcopenia.

Critical outcomes in hospitalized patients with cirrhosis include in-hospital mortality, length of stay (LoS), cost of hospitalization (CoH) and discharge disposition16. Sarcopenia in cirrhosis adversely affects these outcome measures [15,16], but the contributions of primary and secondary sarcopenia in aging patients with cirrhosis is not known. Published reports use ICD codes wherein the term sarcopenia has been used only since the transition to ICD-10 and was intended to code for primary sarcopenia of aging [21]. Since restricting ICD codes in this study would limit our ability to identify the true number of patients with muscle loss [16,22], we have used a previously reported group of codes to define a “muscle loss phenotype” in order to evaluate the consequences of sarcopenia in hospitalized patients [16,23].

With aging, functional decline occurs and hospitalization delays recovery of activity [4,24]. Hospitalization results in more frequent non-home discharge, including institutionalization, in patients with cirrhosis and especially in those with muscle loss phenotype. The impact of muscle loss on discharge disposition in elderly patients with cirrhosis is, however, not known. Interestingly, hospitalized patients with cirrhosis have a greater prevalence of muscle loss phenotype and worse outcomes than an older hospitalized general medicine population (GMP) [16,25]. Increasing age was a risk factor for adverse outcomes, but whether there are systematic differences in the prevalence of sarcopenia in cirrhosis with aging compared to the GMP and if the outcomes were different in different age strata in patients with cirrhosis with and without a muscle loss phenotype have not been reported. 

Data in hospitalized adults with cirrhosis show that a muscle loss phenotype is more frequent in patients with cirrhosis and is an independent risk factor for in-hospital mortality and increased LoS and CoH per admission [16]. There are no data on the prevalence or consequences of sarcopenia in an age-stratified hospitalized cirrhotic population. Older patients with cirrhosis are likely to have compound sarcopenia, while the younger patients with cirrhosis have only secondary sarcopenia. To study the impact of aging on cirrhosis-related secondary sarcopenia, the prevalence and adverse consequences of a muscle loss phenotype in GMP and patients with cirrhosis were evaluated in the Nationwide Inpatient Sample (NIS) database for the years 2010–2014. We tested our hypothesis that muscle loss phenotype is more frequent and is associated with worse clinical outcomes in hospitalized patients with cirrhosis who are older compared to those who are younger. For each age stratum, we also hypothesize that patients with cirrhosis and muscle loss phenotype have worse outcomes than those without a muscle loss phenotype or GMP in the same age stratum. 

## 2. Materials and Methods

The NIS database is maintained by the Agency for Healthcare Research and Quality via the Healthcare Cost and Utilization Project (HCUP). The data organizations that contribute to this project are listed at https://www.hcup-us.ahrq.gov/db/hcupdatapartners.jsp (accessed 23 June 2020). A data user agreement was signed with the Agency for Healthcare Research and Quality. Administrative data from nearly 8 million annual hospitalizations from about 1000 U.S. hospitals including academic medical centers and public hospitals (non-federal) are hosted in the NIS and include primary and secondary diagnoses, patient demographics, hospital LoS, discharge status, in-hospital mortality, CoH, and severity/comorbidity measures. Readmissions and individual patient identifying information including specific laboratory values are not available. Patients were stratified into 3 categories based on age at admission: ≤50 years old, 51–65 years old and >65 years old. The cutoff of 65 years old was chosen to define the elderly population as it is the age that most individuals in the United States are eligible for retirement benefits and is used by the Center for Medicare and Medicaid Services to allow for transition to federally guaranteed healthcare beyond employer provided medical insurance. 

Information from the NIS database was aggregated for the dates between 1 January 2010 and 31 December 2014 to capture 5 years of consistent, encounter-level hospitalization data before ICD-10 codes were introduced in 2015. It has been reported that a narrow use of ICD-9 codes may unintentionally omit data from patients who do in fact have muscle loss [16]. In the ICD-9 list of codes, there is no single unique code for muscle loss; so as previously reported [16], we defined a composite term of a “muscle loss phenotype” that comprises multiple ICD-9 codes that together define the condition of loss of muscle or contractility. Some of the codes we selected included “malnutrition of moderate degree” (263.0) and “cachexia” [799.4], as examples. While an individual code for “sarcopenia” was introduced in 2015 with ICD-10, it was intended and has been interpreted clinically as a code for primary sarcopenia, or sarcopenia of aging. However, with the increasing recognition in the clinical community of secondary sarcopenia of chronic disease, we anticipate the interpretation of the code for sarcopenia will broaden with time and should be used in future analyses. 

Hospitalizations for maternal/pregnancy care were excluded using the Major Diagnostic Category 14: “Pregnancy, Childbirth and the Puerperium” codes. In addition to the standard terminology for race/ethnicity, we included a category termed “Other” which includes Asian/Pacific Islanders and Native Americans. Elixhauser co-morbidity indices, which predict in-hospital mortality and discharge disposition based on the presence or absence of certain co-morbidities, were calculated from the NIS datasets using the relevant ICD-9 codes [16,26]. A random sample of 2% of hospitalized GMP was included to determine if this cohort was different from that in the cirrhosis group and to ensure an approximately 5:1 ratio of GMP to cirrhosis patient population. The overall, but not age stratified characteristics, of this population have been previously reported [16]. The total number differ from our previous publication because we excluded variables with missing values as opposed to utilizing simple imputation for missing values. 

Additional outcomes analyzed included the LoS, CoH, in-hospital mortality, and discharge disposition. For these analyses, we used our previously reported terms: “routine discharge” for patients sent home from the hospital without any assistance and “non-routine discharge” or release to home with any home health aide/support, to a nursing home, to a rehabilitation facility, or against medical advice [16,23]. Non-routine discharge was considered an undesirable outcome because it is recommended for patients with functional limitations and correspondingly, in these analyses, “non-routine discharge” was used as a surrogate for impaired functional status at discharge, as previously reported [16,23]. To evaluate differences among patient outcomes and clinical characteristics in various geographies and socioeconomic statuses, the location of the hospital and insurance carrier types were also analyzed.

Statistical analysis: The primary outcome was in-hospital mortality across age strata in patients with cirrhosis with or without a muscle loss phenotype. Other outcomes of interest included the LoS and CoH for each admission across age strata. Summary statistics, median values, multivariate logistic and linear regression analyses and model fit were performed as previously described [16,23]. A confidence level of 95% was used for all intervals unless otherwise noted. Covariates known to affect risk for muscle loss phenotype were included in our multivariate regression model as previously reported [16,23]. A linear regression analysis of the Elixhauser comorbidity score as the dependent variable was also performed. Statistical analyses were performed using R version 4.0.0 (The R Foundation for Statistical Computing, Vienna, Austria) and SAS 9.4 software (SAS Institute, Cary, NC, USA). Our study and its methods were in accordance to the Strengthening the Reporting of Observational Studies in Epidemiology (STROBE) guidelines [27]. The participant flow chart that conforms to the CONSORT statement is shown in Supplementary Figure S1. 

## 3. Results

**Figure 1:**
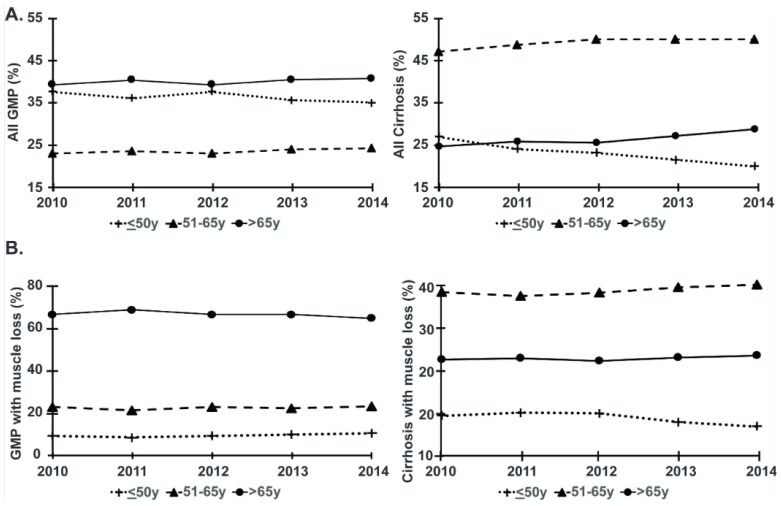
Increasing proportion of older patients with cirrhosis with and without muscle loss phenotype. (**A**) Line graphs depicting percentage of the analyzed random 2% inpatient sample of the general medical population (GMP) and hospitalized patients with cirrhosis from the National Inpatient Sample (NIS) database each year from 2010–2014 who were ≤50, 50–64, and >65 years old. (**B**) Line graphs depicting percentage of the analyzed inpatient sample of those GMP or patients with cirrhosis with muscle loss phenotype each year from the NIS from 2010–2014 who were ≤50, 50–64, and >65 years old.

The overall clinical, demographic characteristics, and outcomes for hospitalized GMP and patients with cirrhosis stratified by age are shown in Table 1, Supplementary Table S1. Between 2010–2014, we evaluated a random 2% sample (*n* = 517,605) of hospitalized GMP and compared it to the total cohort of hospitalized patients with cirrhosis from the same time period (*n* = 106,835). After stratification of subjects into three age groups (≤50 years old, 51–65 years old and >65 years old), we found that the prevalence of Black race and Hispanic ethnicity were lower in older inpatients and that there were more female than male cirrhotic inpatients in each age category of patients with cirrhosis (Supplementary Figure S2). During the study period, the proportion of all GMP or those with muscle loss phenotype in the different age strata did not change significantly (Figure 1A,B). However, over the years 2010 to 2014, there was an increase in both the proportion of hospitalized patients >65 years old with cirrhosis with and without muscle loss phenotype (Figure 1A) as well as that of inpatients with cirrhosis aged 51–65 years old and >65 years old with a muscle loss phenotype (Figure 1B). The mean age of GMP ≤50 years old was significantly lower, while the mean age of GMP >65 years old was significantly higher (*p* < 0.001 for both), than that of matched age strata for hospitalized patients with cirrhosis. In-hospital mortality and muscle loss phenotype nearly doubled for the older age groups within both the GMP and cirrhotic patients. In younger patients with cirrhosis, LoS, in-hospital mortality, CoH per admission, muscle loss phenotype, number of diagnoses on discharge, and Elixhauser score were significantly higher (*p* < 0.001) when compared to older GMP. In addition, all patients with cirrhosis (compared to the GMP within the same age group) and older GMP (compared to younger GMP) were more likely to require transfer to a skilled nursing facility or require home health care. The prevalence of comorbidities including alcohol abuse and diabetes was significantly higher in patients with cirrhosis in each age stratum compared to that in GMP. However, hypertension was significantly higher (*p* < 0.001) in patients with cirrhosis aged ≤50 years old compared to GMP aged ≤50 years old, while in the higher age strata, hypertension was less frequent in hospitalized patients with cirrhosis compared to similar aged GMP (Table 1). 

The clinical and demographic characteristics of hospitalized GMP and cirrhosis patients with muscle loss phenotype grouped by age are shown in Table 2, Supplementary Table S2, and Supplementary Figure S3. We noted that patients with cirrhosis with muscle loss had significantly higher (*p* < 0.001) in-hospital mortality (across all age groups) and CoH (in the 51–65 and >65 year-old age strata) than comparable GMP in the same age strata. Similarly, in-hospital mortality was significantly higher (*p* < 0.001) in younger patients with cirrhosis with muscle loss compared to both younger and older GMP with or without muscle loss. In patients with cirrhosis, older age with muscle loss phenotype resulted in a lower likelihood of discharge to home than the younger population of patients with cirrhosis or the equivalent GMP age group with muscle loss phenotype. Alcohol abuse disorder was significantly higher (*p* < 0.001) in patients with cirrhosis with muscle loss than GMP with muscle loss for each comparable age group. Prevalence of comorbidities as a total percentage, Elixhauser score, and alcohol abuse were significantly higher (*p* < 0.001) in patients with cirrhosis than in GMP with muscle loss phenotype in each of the age strata (Table 2).

Regression analysis comparing hospitalized GMP and patients with cirrhosis with muscle loss phenotype showed that CoH per admission, in-hospital mortality, and LoS were significantly higher in patients with cirrhosis 51–65 years old compared to GMP of the same age group, even after adjustment for covariables and other demographic features. Among patients with cirrhosis with muscle loss phenotype, CoH per admission and in-hospital mortality were higher than in GMP with muscle loss phenotype for those >65 years old, while LoS was not significantly different. In-hospital mortality was higher for the ≤50 year-old patient population with cirrhosis with muscle loss phenotype when compared to GMP with muscle loss phenotype of the same age group, while CoH and LoS were not different (Table 3). 

Clinical, demographic characteristics, and outcomes of GMP with and without muscle loss phenotype grouped by age are shown in Table 4, Supplementary Table S3 and Supplementary Figure S4. The proportion of hospitalized GMP males with muscle loss phenotype was significantly higher than that without muscle loss phenotype. Muscle loss phenotype was also associated with significantly higher (*p* < 0.001) in-hospital mortality, LoS, CoH per admission, and Elixhauser comorbidity score for all age groups of GMP. With increasing age, the GMP discharge-to-home rate was lower in those with muscle loss phenotype compared to those without. In-hospital mortality nearly doubled with increasing age group among GMP without muscle loss phenotype yet was significantly higher still (*p* < 0.001) in each respective GMP age group with muscle loss phenotype. In-hospital mortality rate and LoS in patients with muscle loss phenotype in GMP ≤50 years old was higher than those >65 years old without muscle loss diagnosis. In addition, muscle loss phenotype was associated with a higher prevalence of alcohol disorder and hypertension in GMP of all age groups. Prevalence of comorbidities including alcohol abuse, diabetes, and hypertension were significantly higher (*p* < 0.001) in GMP ≤50 years old with muscle loss phenotype compared to those ≤50 years old without muscle loss phenotype for each age group (Table 4). 

The clinical and demographic characteristics of hospitalized patients with cirrhosis with and without muscle loss phenotype stratified by age classes are shown in Table 5, Supplementary Table S4, and Supplementary Figure S5. There was a male preponderance among hospitalized patients with cirrhosis ≤50 years old with muscle loss. However, there was no difference in sex distribution of patients with cirrhosis in the 51–65 and >65 year-old age groups with or without a muscle loss phenotype. Muscle loss phenotype in patients with cirrhosis was associated with significantly higher (*p* < 0.001) in-hospital mortality, LoS, and CoH per admission. Patients with cirrhosis and a muscle loss phenotype also had a significantly lower (*p* < 0.001) likelihood of discharge home in each age stratum. Similar to our observations in the GMP, the mortality rate in patients with cirrhosis ≤50 years old with a muscle loss phenotype was higher than that in patients with cirrhosis >65 years old without a diagnosis of muscle loss. In matched comparisons of each of the age strata, there was a significantly higher CoH in patients with cirrhosis and muscle loss phenotype compared to those without muscle loss phenotype. Individual comorbidities as well as the composite Elixhauser index were significantly higher (*p* < 0.001) in patients with cirrhosis and a muscle loss phenotype compared to those without (Table 5). 

A multivariate linear regression model of the Elixhauser comorbidity score comparing age categories and muscle loss phenotype in hospitalized patients with cirrhosis and GMP is shown in Supplementary Table S5. The Elixhauser comorbidity score in patients with cirrhosis was significantly higher in the older age categories. Among patients with cirrhosis, the adjusted comorbidity score was 1.79 points higher (95% CI 1.61–1.96, *p* < 0.001) for those 51–65 years old and 5.43 points higher (95% CI 5.24–5.62, *p* < 0.001) for those >65 years old as compared to the ≤50 year-old group. For those with muscle loss phenotype, the Elixhauser score for patients with cirrhosis was 14.15 points higher (95% CI 13.95–14.35, *p* < 0.001) when compared to those without muscle phenotype even after adjustment for age category. 

A multivariate linear regression model comparing age categories and muscle loss phenotype in the GMP showed that the Elixhauser comorbidity score was also significantly higher in those 51–65 years old (2.93, 95% CI: 2.87–2.99, *p* < 0.001) and in those ≥65 years old (5.94, 95% CI: 5.89–5.99, *p* < 0.001) compared to those aged ≤50 years old. Muscle loss phenotype was similarly associated with a significantly higher Elixhauser comorbidity score in the GMP population after adjustment for age category (16.52 points, CI: 16.37–16.68, *p* < 0.001). A multivariate linear regression model comparing the Elixhauser comorbidity score in those with cirrhosis versus GMP, adjusted for age (as a continuous variable), showed that patients with cirrhosis had a significantly higher comorbidity score by 10.57 points (CI: 10.51–10.64, *p* < 0.001) compared to the GMP. Specifically, for each increase in age by 1 year, the comorbidity score increased by 0.145 points even when adjusted for the presence of cirrhosis (95% CI: 0.144–0.146, *p* < 0.001). 

Multivariate analyses, predicting variables associated with increased in-hospital mortality, LoS and CoH per admission in different age groups of hospitalized patients with cirrhosis (Supplementary Tables S6–S8), showed that muscle loss phenotype, chronic lung disease, coagulopathy, congestive heart failure and acute kidney injury were independently associated with increased LoS for all age groups. Alcohol abuse was associated with decreased LoS in all age groups among patients with cirrhosis. Muscle loss phenotype, metastatic cancer, solid tumors, coagulopathy, anemia, congestive heart failure, diabetes (complicated), and acute kidney injury were associated with increased CoH for all age groups, while alcohol abuse was associated with decreased CoH. Muscle loss phenotype, metastatic cancer, solid tumors, coagulopathy, congestive heart failure, diabetes mellitus, and acute kidney injury were also associated with increased risk for in-hospital mortality. Black race was associated with increased in-hospital mortality when compared to White race, but LoS and CoH were not significantly different when race/ethnicities were compared across all age groups. 

## 4. Discussion

Our analyses of a large inpatient dataset show that over a 5-year period, there was an increase in the proportion of hospitalized patients with cirrhosis >65 years old. Aging increased critical outcomes evaluated in this study: LoS, CoH per admission and in-hospital mortality in both the GMP and those with cirrhosis; however, each of these outcomes was worse in patients with cirrhosis for each age stratum. The proportion of muscle loss phenotype increased with increasing age strata, but to a greater extent in patients with cirrhosis than in the GMP. Muscle loss phenotype in both the GMP and patients with cirrhosis was associated with higher inpatient mortality, LoS, and CoH, but each outcome was worse in patients with cirrhosis than in the GMP. These data demonstrate that the population of hospitalized older adults with cirrhosis continues to increase and that cirrhosis in older adults is associated more frequently with a muscle loss phenotype and adverse consequences than in younger patients with cirrhosis or age strata matched GMP.

There are increasing data that the population of patients with chronic liver disease continues to increase both in the United States and globally [6,8,28,29,30]. However, there are no published data on muscle loss phenotype or sarcopenia in older patients with cirrhosis. Primary sarcopenia is one of the recognized consequences of aging and, consistently, our data in hospitalized GMP and adults with cirrhosis show an aging-related increase in prevalence of a muscle loss phenotype in both groups. However, the higher frequency of muscle loss phenotype in cirrhosis for each stratum shows that an additional element of secondary sarcopenia contributes to the primary sarcopenia of aging, resulting in compound sarcopenia that occurs in patients with cirrhosis and potentially those with other chronic diseases as they age. These data are consistent with previous reports that malnutrition, of which the major component is muscle loss, was more frequent in hospitalized adults with cirrhosis than comparable GMP [16,25]. Our observations show a high prevalence and worse clinical consequences of the presence of a muscle loss phenotype in each of the identified age strata in hospitalized patients with cirrhosis compared to the GMP. 

Most published data on aging patients with cirrhosis are derived from LT recipient databases [8,30,31,32,33]. In the United States, the mean age of transplant recipients increased from 29 years old to 45 years old between 1985 and 1995, while the proportion of LT recipients aged 65 years and older increased from 9% in 2002 to nearly 20% in 20178. The mean age of patients listed for LT increased from 51.2 years old in 2002 to 55.7 years old in 2014. Similar observations have been reported in the European transplant data [8]. Consistent with reports of higher transplant waitlist mortality in older patients with cirrhosis [32,34], in the present study, the oldest age strata of patients with cirrhosis had worse clinical outcomes, including higher inpatient mortality, than younger patients with cirrhosis and age strata matched GMP. Despite the increase in the number of older patients with cirrhosis, potential reasons for lower transplant listings and worse post-transplant outcomes in the elderly include greater comorbidities including cardiovascular, renal, and metabolic bone disease and malignancies [35,36,37,38]. Consistently, in the present study, older patients with cirrhosis had more comorbidities than younger patients with cirrhosis. Additionally, both the GMP and patients with cirrhosis with a muscle loss phenotype had more comorbidities than those without a muscle loss phenotype. These data are similar to those reported in a much smaller cohort from the Indiana Health system in which the aging patient population with cirrhosis was more frequently associated with mortality [29], but the impact of muscle loss phenotype on mortality was not reported. 

Others have shown that frailty, or loss of physical function driven primarily by muscle loss, is associated with increased mortality [34,39]. Frailty, determined by measures of contractile function, in hospitalized patients with cirrhosis is associated with poor clinical outcomes [40]. There are currently no ICD (9 or 10) codes for frailty or loss of physical function and comparisons of frailty and its adverse impact in different age strata have not been reported. While the concept of frailty was developed in the aging population, chronic diseases (including cirrhosis) have been reported to have accelerated senescence [41,42] with measures of impaired physical function. Even though a muscle loss phenotype is generally accompanied by contractile dysfunction, the underlying mechanisms of physical frailty are not as well identified as they are for sarcopenia. There are emerging data that contractile dysfunction may be due to post-translational modification of contractile proteins and mitochondrial dysfunction [43,44]. These data suggest that sarcopenia and frailty may have common underlying mechanisms. Unlike the sarcopenic phenotype that has been well defined in cirrhosis, there is a need to clarify the interactions and consequences of the frailty phenotype [45]. Our observations also show that hospitalized younger patients with cirrhosis without a muscle loss phenotype had clinical outcomes similar to those of older GMP, suggesting functional senescence in cirrhosis. This is further supported by our analyses that a significantly higher proportion of patients with cirrhosis in each age stratum had a muscle loss phenotype compared to those in comparable age strata among the GMP. 

Our observations that comorbidities are more frequent in both GMP and patients with cirrhosis with a muscle loss phenotype are consistent with and extend earlier reports that other complications of liver disease are more common in sarcopenic patients with cirrhosis [46], and shows the adverse impact of “compound sarcopenia” in older patients with cirrhosis. It is not clear if comorbidities contribute to the muscle loss phenotype, whether patients with muscle loss have more comorbidities, or if there is a bidirectional interaction between these two adverse clinical consequences and these questions need to be evaluated in future studies. Sarcopenia in cirrhosis adversely affects outcomes, and since compound sarcopenia occurs in older patients with cirrhosis, adverse consequences are likely to be worse in such patients. There are few studies on outcomes and, specifically, responses to hospitalization in older patients with cirrhosis who have more comorbidities [47,48]. Even though primary sarcopenia adversely impacts outcomes, our prespecified outcomes in hospitalized patients with cirrhosis ≤50 years old with a muscle loss phenotype are worse than older GMP. These observations support an accelerated aging phenotype in patients with cirrhosis and that muscle loss of aging compounds the disease-related sarcopenia of cirrhosis [41,42], contributing to the high proportion of older patients with cirrhosis with a muscle loss phenotype (Graphical abstract). 

Graphical abstract: Aging related primary sarcopenia compounds secondary sarcopenia of cirrhosis with worse clinical outcomes. 

Since careful characterization of older patients with cirrhosis is critical to optimize management strategies, including the decision for or against transplantation, evaluation of the presence of a muscle loss phenotype will help identify appropriate candidates for LT within this group. Since the population of older patients with cirrhosis and those receiving LT continues to increase [8], the presence of a muscle loss phenotype is likely to have a greater impact on long-term outcomes because, unlike other complications of liver disease, sarcopenia does not improve after LT [13,49]. Whether post-transplant sarcopenia is worse in the older transplant recipient is not well described, primarily because it is only recently that older patients with cirrhosis are increasingly being transplanted [42]. Similar to the approaches in younger patients with cirrhosis, aggressive strategies to control muscle loss are required to improve outcomes both before and after LT. Immunosuppressive medications are a potential reason for post-LT muscle loss [49]. Lower tolerance to immunosuppressive medications in older patients with cirrhosis may contribute to greater post LT sarcopenia, but this has not been evaluated to date. Post-transplant sarcopenia is associated with other components of metabolic syndrome and adverse clinical outcomes that may be more frequent in older patients with cirrhosis [49,50,51]. The present studies lay the foundation for evaluating the risks for the development and worsening of muscle loss after LT in older patients with cirrhosis and its adverse consequences. 

We and others had projected an increase in alcohol related liver disease and cirrhosis in the coming decade [52]. Interestingly, the proportion of older patients with cirrhosis and alcohol use disorder is less than in younger patients and these data are similar to that reported by others [29]. Potential reasons for lower rates of alcohol use disorder in older patients with cirrhosis include mortality from complications related to alcohol consumption [52], higher mortality in alcoholic cirrhosis compared to other etiologies of liver disease, and an increase in the prevalence of non-alcoholic fatty liver disease-related cirrhosis with age [29,53]. Among both the GMP and patient population with cirrhosis, we noted a lower proportion of Black patients in the older age strata, which may be due to a higher mortality rate in this racial group [54].

Our data show an increase over time in the number of older hospitalized patients with cirrhosis; additionally, in the older age strata, the prevalence of muscle loss phenotype is nearly double that in younger patients with cirrhosis. Our observation of an independent adverse effect of a muscle loss phenotype on clinical outcomes that increases with aging reveals the urgent need to target and manage sarcopenia, specifically in older adults with cirrhosis.

## Figures and Tables

**Table 1:** Demographic characteristics for hospitalized general medicine and patients with cirrhosis, grouped by age.

	GMP			Patients with Cirrhosis		
Age Categories (Years)	≤50	51–65	>65	≤50	51–65	>65
Number of patients	188,510	121,829	207,266	24,848	52,969	29,018
Female (%)	131,500 (69.8) ^c,f^	60,668 (49.8) ^c,i^	117,519 (56.7) ^f,i^	15,682 (63.1) ***^,c,f^	34,978 (66.0) ***^,c,i^	15,618 (53.8) ***^,f,i^
Age (mean (SD))	34.1 (9.4)	58.1 (4.3)	78.1 (7.8)	43.2 (6.4) ***	57.3 (4.1) ***	74.1 (6.5) ***
Race (%)						
White	108,484 (57.5) ^c,f^	83,825 (68.8) ^c,i^	162,751 (78.5) ^f,i^	16,372 (65.9) ***^,c,f^	36,330 (68.6) ^c,i^	21,447 (73.9) ***^,f,i^
Black	36,259 (19.2) ^c,f^	21,769 (17.9) ^c,i^	21,446 (10.3) ^f,i^	2195 (8.8) ***^,c,e^	5708 (10.8) ***^,c,i^	2005 (6.9) ***^,e,i^
Hispanic	29,665 (15.7) ^c,f^	10,262 (8.4) ^c,i^	13,603 (6.6) ^f,i^	4592 (18.5) ***^,c,f^	8230 (15.5) ***^,c,i^	3946 (13.6) ***^,f,i^
Others	14,102 (7.5) ^c,f^	5973 (4.9) ^c,i^	9466 (4.6) ^f,i^	1689 (6.8) ***^,c,f^	2701 (5.1) ^c,g^	1620 (5.6) ***^,g,i^
LoS (mean (SD))	3.8 (5.7) ^c,f^	5.1 (6.90) ^c,i^	5.4 (6.6) ^f,i^	7.7 (10.8) ***^,c,f^	8.1 (10.0) ***^,c,i^	8.2 (8.6) ***^,f,i^
In-hospital mortality (%)	948 (0.5) ^c,f^	2393 (2.0) ^c,i^	8011 (3.9) ^f,i^	1974 (7.9) ***^,c,f^	5121 (9.7) ***^,c,i^	3362 (11.6) ***^,f,i^
CoH in USD (median (IQR))	4768.0 (2979.0, 8367.0) ^c,f^	8197.0 (4664.0, 14,755.0) ^c,i^	8007.00 (4735.0, 14,121.0) ^f,i^	9631.8 (5610.2, 19,696.6) ***^,c,f^	10,643.2 (6000.5, 22,137.5) ***^,c,i^	11,408.4 (6569.9, 21,598.4) ***^,f,i^
Muscle loss phenotype (%)	1058 (0.6) ^c,f^	2417 (2.0) ^c,i^	7052 (3.4) ^f,i^	2511 (10.1) ***^,c,f^	6688 (12.6) ***^,c,i^	4501 (15.5) ***^,f,i^
Number of diagnoses on discharge (mean (SD))	6.7 (4.5) ^c,f^	10.4 (5.6) ^c,i^	12.3 (5.7) ^f,i^	15.5 (7.2) ***^,c,f^	17.5 (7.5) ***^,c,i^	20.1 (7.2) ***^,f,i^
Elixhauser score (mean (SD))	0.8 (6.4) ^c,f^	3.9 (9.0) ^c,i^	7.2 (9.7) ^f,i^	12.1 (11.6) ***^,c,f^	14.3 (12.3) ***^,c,i^	18.3 (12.9) ***^,f,i^
Comorbidities (%)						
Acute kidney injury	6075 (3.2) ^c,f^	13,329 (10.9) ^c,i^	40,755 (19.7) ^f,i^	2948 (11.9) ***^,c,f^	10,964 (20.7) ***^,c,i^	10,912 (37.6) ***^,f,i^
Alcohol abuse	9879 (5.2) ^c,f^	8494 (7.0) ^c,i^	3863 (1.9) ^f,i^	16,556 (66.6) ***^,c,f^	28,710 (54.2) ***^,c,i^	7072 (24.4) ***^,f,i^
Diabetes (uncomplicated)	13,969 (7.4) ^c,f^	29,136 (23.9) ^c,i^	53,496 (25.8) ^f,i^	3888 (15.6) ***^,c,f^	13,733 (25.9) ***^,c,i^	10,911 (37.6) ***^,f,i^
Diabetes (complicated)	3597 (1.9) ^c,f^	7667 (6.3) ^c,i^	11,664 (5.6) ^f,i^	979 (3.9) ***^,c,f^	4481 (8.5) ***^,c,i^	3486 (12.0) ***^,f,i^
Hypertension	36,017 (19.1) ^c,f^	71,179 (58.4) ^c,i^	145,906 (70.4) ^f,i^	8017 (32.3) ***^,c,f^	25,695 (48.5) ***^,c,i^	18,740 (64.6) ***^,f,i^
Home discharge	166,183 (88.7) ^c,f^	84,046 (70.4) ^c,i^	92,455 (46.4) ^f,i^	16,764 (73.4) ***^,c,f^	29,707 (62.2) ***^,f,i^	10,258 (40.1) ***^,f,i^

Abbreviations: CoH: Cost of hospitalization, GMP: general medical population, LoS: Length of stay, SD: Standard deviation, USD: US dollars. GMP vs. patients with cirrhosis between each age group: *** *p* < 0.001. Within disease group, ≤50 vs. 51–65: ^c^: *p* < 0.001. Within disease group, ≤50 vs. >65: ^e^: *p* < 0.01; ^f^: *p* < 0.001. Within disease group, 51–65 vs. >65: ^g^: *p* < 0.05; ^i^: *p* < 0.001.

**Table 2:** Demographic characteristics of hospitalized general medicine population and patients with cirrhosis with muscle loss phenotype, grouped by age.

	GMP with Muscle Loss Phenotype			Patients with Cirrhosis with Muscle Loss Phenotype		
Age categories (years)	≤50	51–65	>65	≤50	51–65	>65
Number of patients	1058	2417	7052	2511	6688	4501
Female (%)	519 (49.1) ^d^	1111 (46.0) ^i^	3749 (53.2) ^d,i^	1386 (55.2) ***^,c^	4377 (65.4) ***^,c,i^	2474 (55.0) ^i^
Age (mean (SD))	40.1 (8.4)	58.7 (4.2)	79.4 (7.8)	43.0 (6.59) ***	57.7 (4.1)	74.5 (6.7) ***
Race (%)						
White	637 (60.2) ^f^	1579 (65.3) ^i^	5274 (74.8) ^f,i^	1751 (69.7) ***	4742 (70.9) ***	3304 (73.4)
Black	230 (21.7) ^f^	533 (22.1) ^i^	942 (13.4) ^f,i^	242 (9.6) ***^,a^	809 (12.1) ***^,a,i^	370 (8.2) ***^,i^
Hispanic	120 (11.3) ^e^	187 (7.7)	471 (6.7) ^e^	320 (12.7)	752 (11.2) ***	531 (11.8) ***
Others	71 (6.7)	118 (4.9)	365 (5.2)	198 (7.9) ^a^	385 (5.8) ^a^	296 (6.6) **
LoS (mean (SD))	18.1 (20.8) ^c,f^	14.5 (15.8) ^c,i^	11.5 (12.2) ^f,i^	15.8 (17.1) ***^,c,f^	14.7 (14.7) ^c,i^	12.3 (11.8) ***^,f,i^
In-hospital mortality (%)	70 (6.6) ^b,f^	276 (11.4) ^b^	834 (11.8) ^f^	444 (17.7) ***^,cf^	1248 (18.7) ***^,c,i^	897 (19.9) ***^,f,i^
CoH in USD (median (IQR))	25,214.5 (11,835.5, 58,475.0) ^c,f^	19,629.0 (10,221.0, 42,480.3) ^c,i^	14,611.0 (8059.0, 28,393.0) ^f,i^	25,506.7 (13,172.4, 52,870.7) ^f^	23,538.8 (12,421.5, 48,347.4) ***^,i^	18,645.07 (10,762.5, 35,458.6) ***^,f,i^
Number of diagnoses on discharge (mean (SD))	21.3 (3.7)	21.4 (3.8)	21.4 (3.6)	24.2 (3.7) ***^,c,f^	24.7 (3.5) ***^,c,i^	25.1 (3.1) ***^,f,i^
Elixhauser score (mean (SD))	19.3 (11.1) ^c,f^	21.4 (11.4) ^c,i^	22.4 (11.0) ^f,i^	25.6 (10.3) ***^,c,f^	26.8 (10.8) ***^,c,i^	29.5 (11.2) ***^,f,i^
Comorbidities (%)	19.3 (11.1) ^c,f^	21.4 (11.4) ^c,i^	22.4 (11.0) ^f,i^	25.6 (10.3) ***^,c,f^	26.8 (10.8) ***^,c,i^	29.5 (11.2) ***^,f,i^
Acute kidney injury	224 (21.2) ^c,f^	588 (24.3) ^c,i^	2331 (33.1) ^f,i^	532 (21.2) ^c,f^	1887 (28.2) ***^,^^c^^,i^	1878 (41.7) ***^,^^f^^,i^
Alcohol abuse	130 (12.3) ^f^	325 (13.4) ^i^	229 (3.2) ^f,i^	1602 (63.8) ***^,c,f^	3709 (55.5) ***^,c,i^	1228 (27.3) ***^,f,i^
Diabetes (uncomplicated)	159 (15.0) ^c,f^	518 (21.4) ^c,h^	1719 (24.4) ^f,h^	378 (15.1) ^c,f^	1551 (23.2) ^c,i^	1374 (30.5) ***^,f,i^
Diabetes (complicated)	109 (10.3)	294 (12.2) ^i^	668 (9.5) ^i^	141 (5.6) ^c,f^	652 (9.7) ***^,c,i^	535 (11.9) ***^,f,i^
Hypertension	411 (38.8) ^c,f^	1367 (56.6) ^c,i^	4864 (69.0) ^f,i^	899 (35.8) ^c,f^	3404 (50.9) ***^,c,i^	2837 (63.0) ***^,f,i^
Home discharge	393 (39.8) ^c,f^	609 (28.5) ^c,i^	906 (14.6) ^f,i^	944 (45.8) ***^,c,f^	1837 (33.8) ***^,c,i^	679 (18.9) ***^,f,i^

Abbreviations: CoH: Cost of hospitalization, GMP: General medical population, LoS: Length of stay, SD: Standard deviation, USD: US dollars. GMP with muscle loss vs. patients with cirrhosis with muscle loss, between each age group: ** *p* < 0.01, *** *p* < 0.001. Within disease group, ≤50 vs. 51–65: ^a^: *p* < 0.05; ^b^: *p* < 0.01; ^c^: *p* < 0.001. Within disease group, ≤50 vs. >65: ^d^: *p* < 0.05; ^e^: *p* < 0.01; ^f^: *p* < 0.001. Within disease group, 51–65 vs. >65: ^h^: *p* < 0.01; ^i^: *p* < 0.001.

**Table 3:** Regression analysis for hospitalized general medical and patients with cirrhosis with muscle loss phenotype.

	Patients with Cirrhosis vs. General Medical Patients Age ≤ 50		Patients with Cirrhosis vs. General Medical Patients Age 51–65		Patients with Cirrhosis vs. General Medical Patients Age > 65	
	Unadjusted OR (95% CI)	Adjusted * OR (95% CI)	Unadjusted OR (95% CI)	Adjusted * OR (95% CI)	Unadjusted OR (95% CI)	Adjusted * OR (95% CI)
Cost of hospitalization	1.00 (0.93, 1.08)	0.92 (0.81, 1.04)	1.20 (1.14, 1.25)	1.24 (1.15, 1.34)	1.26 (1.22, 1.31)	1.10 (1.04, 1.17)
Hospital mortality	2.89 (2.06–4.17)	2.15 (1.44–3.27)	1.98 (1.63, 2.43)	1.86 (1.49–2.33)	1.87 (1.62–2.16)	1.77 (1.50–2.08)
Length of stay	0.94 (0.89, 1.00)	0.91 (0.82, 1.01)	1.05 (1.01, 1.09)	1.10 (1.03, 1.16)	1.09 (1.06, 1.12)	1.02 (0.98, 1.07)

OR: Odds ratio, CI: Confidence interval. * Adjusted for sex, race, and comorbidities (acute kidney injury, congestive heart failure, anemia, chronic lung disease, alcohol abuse, coagulopathy, aids, metastatic cancer, cancer with solid tumors, diabetes (uncomplicated), diabetes (complicated).

**Table 4:** Demographic characteristics of hospitalized general medicine patients with/without muscle loss phenotype grouped by age.

	GMP without Muscle Loss Phenotype			GMP with Muscle Loss Phenotype		
Age categories (years)	≤50	51–65	>65	≤50	51–65	>65
Number of patients	187,452	119,412	200,214	1058	2417	7052
Female (%)	130,981 (69.9) ^c,f^	59,557 (49.9) ^c,i^	113,770 (56.8) ^f,i^	519 (49.1) ***^,d^	1111 (46.0) ***^,i^	3749 (53.2) ***^,d,i^
Age (mean (SD))	34.1 (9.3)	58.1 (4.3)	78.0 (7.8)	40.1 (8.4) ***	58.7 (4.2) ***	79.4 (7.8) ***
Race (%)						
White	108,484 (57.5) ^c,f^	83,825 (68.8) ^c,i^	162,751 (78.5) ^f,i^	637 (60.2)	1579 (65.3) ***	5274 (74.8) ***
Black	36,259 (19.2) ^c,f^	21,769 (17.9) ^c,i^	21,446 (10.3) ^f,i^	230 (21.7) *^,a^	533 (22.1) ***^,a,i^	942 (13.4) ***^,i^
Hispanic	29,665 (15.7) ^c,f^	10,262 (8.4) ^c,i^	13,603 (6.6) ^f,i^	120 (11.3) ***	187 (7.7)	471 (6.7)
Others	14,102 (7.5) ^c,f^	5973 (4.9) ^c,i^	9466 (4.6) ^f,i^	71 (6.7) ^a^	118 (4.9) ^a^	365 (5.2) *
LoS (mean (SD))	3.7 (5.3) ^c,f^	4.9 (6.5) ^c,i^	5.2 (6.2) ^f,i^	18.1 (20.8) ***^,c,f^	14.5 (15.8) ***^,c,i^	11.5 (12.2) ***^,f,i^
In-hospital mortality (%)	878 (0.5) ^c,f^	2117 (1.8) ^c,i^	7177 (3.6) ^f,i^	70 (6.6) ***^c,f^	276 (11.4) ***^c,i^	834 (11.8) ***^f,i^
CoH in USD (median (IQR))	4746.0 (2971.0, 8284.0) ^c,f^	8080.0 (4622.0, 14,465.5) ^c,i^	7871.0 (4672.5, 13,787.0) ^f,i^	25,214.5 (11,835.5, 58,475.0) ***^,c,f^	19,629.0 (10,221.0, 42,480.3) ***^,c,i^	14,611.0 (8059.0, 28,393.0) ***^,f,i^
Number of diagnoses on discharge (mean (SD))	6.6 (4.4) ^c,f^	10.2 (5.4) ^c,i^	12.0 (5.5) ^f,i^	21.3 (3.7) ***	21.4 (3.8) ***	21.4 (3.6) ***
Elixhauser score (mean (SD))	0.7 (6.2) ^c,f^	3.6 (8.6) ^c,i^	6.6 (9.2) ^f,i^	19.3 (11.1) ***^,c,f^	21.4 (11.4) ***^,c,i^	22.4 (11.0) ***^,f,i^
Comorbidities (%)	0.7 (6.2) ^c,f^	3.6 (8.6) ^c,i^	6.6 (9.2) ^f,i^	19.3 (11.1) ***^,c,f^	21.4 (11.4) ***^,c,i^	22.4 (11.0) ***^,f,i^
Acute kidney injury	5851 (3.1) ^c,f^	12,741 (10.7) ^c,i^	38,424 (19.2) ^f,i^	224 (21.2) ***^,f^	588 (24.3) ***^,i^	2331 (33.1) ***^,f,i^
Alcohol abuse	9749 (5.2) ^c,f^	8169 (6.8) ^c,i^	3634 (1.8) ^f,i^	130 (12.3) ***^,f^	325 (13.4) ***^,i^	229 (3.2) ***^,f,i^
Diabetes (uncomplicated)	13,810 (7.4) ^c,f^	28,618 (24.0) ^c,i^	51,777 (25.9) ^f,i^	159 (15.0) ***^,c,f^	518 (21.4) **^,c,h^	1719 (24.4) **^,f,h^
Diabetes (complicated)	3488 (1.9) ^c,f^	7373 (6.2) ^c,i^	10,996 (5.5) ^f,i^	109 (10.3) ***	294 (12.2) ***^,i^	668 (9.5) ***^,i^
Hypertension	35,606 (19.0) ^c,f^	69,812 (58.5) ^c,i^	141,042 (70.4) ^f,i^	411 (38.8) ***^,c,f^	1367 (56.6) ^c,i^	4864 (69.0) *^,f,i^
Home discharge	165,790 (88.9) ^c,f^	83,437 (71.2) ^c,i^	91,549 (47.5) ^f,i^	393 (39.8) ***^,c,f^	609 (28.5) ***^,c,i^	906 (14.6) ***^,f,i^

Abbreviations: CoH: Cost of hospitalization, GMP: General medical population, LoS: Length of stay, SD: Standard deviation, USD: US dollars. GMP without muscle loss vs. GMP with muscle loss phenotype, between each age group: * *p* < 0.05, ** *p* < 0.01, *** *p* < 0.001. Within phenotype group, ≤50 vs. 51–65: ^a^: *p* < 0.05; ^c^: *p* < 0.001. Within phenotype group, ≤50 vs. >65: ^d^: *p* < 0.05; ^f^: *p* < 0.001. Within phenotype group, 51–65 vs. >65: ^h^: *p* < 0.01; ^i^: *p* < 0.001.

**Table 5:** Demographic characteristics of hospitalized patients with cirrhosis with/without muscle loss phenotype, grouped by age.

	Patients with Cirrhosis without Muscle Loss Phenotype			Patients with Cirrhosis with Muscle Loss Phenotype		
Age categories (years)	≤50	51–65	>65	≤50	51–65	>65
Number of patients	22,337	46,281	24,517	2511	6688	4501
Female (%)	14,296 (64.0) ^c,f^	30,601 (66.1) ^c,i^	13,144 (53.6) ^f,i^	1386 (55.2) ***^c^	4377 (65.4) ^c,i^	2474 (55.0) ^i^
Age (mean (SD))	43.2 (6.4)	57.3 (4.1)	74.1 (6.5)	43.0 (6.6)	57.7 (4.1) ***	74.5 (6.7) ***
Race (%)						
White	16,372 (65.9) ^c,f^	36,330 (68.6) ^c,i^	21,447 (73.9) ^f,i^	1751 (69.7) ***	4742 (70.9) ***	3304 (73.4)
Black	2195 (8.8) ^c,e^	5708 (10.8) ^c,i^	2005 (6.9) ^e,i^	242 (9.6) ^a^	809 (12.1) **^,a,i^	370 (8.2) **^,i^
Hispanic	4592 (18.5) ^c,f^	8230 (15.5) ^c,i^	3946 (13.6) ^f,i^	320 (12.7) ***	752 (11.2) ***	531 (11.8) **
Others	1689 (6.8) ^c,f^	2701 (5.1) ^c,g^	1620 (5.6) ^g,i^	198 (7.9) *^,a^	385 (5.8) *^,a^	296 (6.6) **
LoS (mean (SD))	6.8 (9.4) ^c,f^	7.2 (8.8) ^c,h^	7.4 (7.7) ^f,h^	15.8 (17.1) ***^,b,f^	14.7 (14.7) ***^,b,i^	12.3 (11.8) ***^,f,i^
In-hospital mortality (%)	1530 (6.8) ^c,f^	3873 (8.4) ^c,i^	2465 (10.1) ^f,i^	444 (17.7) ***	1248 (18.7) ***	897 (19.9) ***
CoH in USD (median (IQR))	8921.3 (5342.8, 16,837.6) ^c^	9680.6 (5621.7, 18,898.9) ^c,i^	10,439.6 (6164.0, 19,323.8) ^i^	25,506.7 (13,172.4, 52,870.7) ***^,c,f^	23,538.8 (12,421.5, 48,347.4) ***^,c,i^	18,645.07 (10,762.5, 35,458.6) ***^,f,i^
Number of diagnoses on discharge (mean (SD))	14.5 (6.8) ^c,f^	16.5 (7.3) ^c,i^	19.2 (7.4) ^f,i^	24.2 (3.7) ***^,c,f^	24.7 (3.5) ***^,c,i^	25.1 (3.1) ***^,f,i^
Elixhauser score (mean (SD))	10.6 (10.7) ^c,f^	12.4 (11.4) ^c,i^	16.2 (12.1) ^f,i^	25.6 (10.3) ***^,c,f^	26.8 (10.8) ***^,c,i^	29.5 (11.2) ***^,f,i^
Comorbidities (%)						
Acute kidney injury	2416 (10.8) ^c,f^	9077 (19.6) ^c,i^	9034 (36.8) ^f,i^	532 (21.2) ***^,c,f^	1887 (28.2) ***^,c,i^	1878 (41.7) ***^,f,i^
Alcohol abuse	14,954 (66.9) ^c,f^	25,001 (54.0) ^c,i^	5844 (23.8) ^f,i^	1602 (63.8) **^,c,f^	3709 (55.5) *^,c,i^	1228 (27.3) ***^,f,i^
Diabetes (uncomplicated)	3510 (15.7) ^c,f^	12,182 (26.3) ^c,i^	9537 (38.9) ^f,i^	378 (15.1) ^c,f^	1551 (23.2) ***^,c,i^	1374 (30.5) ***^,f,i^
Diabetes (complicated)	838 (3.8) ^c,f^	3829 (8.3) ^c,i^	2951 (12.0) ^f,i^	141 (5.6) ***^,c,f^	652 (9.7) ***^,c,i^	535 (11.9) ^f,i^
Hypertension	7118 (31.9) ^c,f^	22,291 (48.2) ^c,i^	15,903 (64.9) ^f,i^	899 (35.8) ***^,c,f^	3404 (50.9) ***^,c,i^	2837 (63.0) *^,f,i^
Home discharge	15,820 (76.1) ^c,f^	27,870 (65.8) ^c,i^	9579 (43.5) ^f,i^	944 (45.8) ***^,c,f^	1837 (33.8) ***^,c,i^	679 (18.9) ***^,f,i^

Abbreviations: CoH: Cost of hospitalization, LoS: Length of stay, USD: US dollars, SD: Standard deviation. Patients with cirrhosis without muscle loss vs. patients with cirrhosis with muscle loss, between each age group: * *p* < 0.05, ** *p* < 0.01, *** *p* < 0.001. Within phenotype group, ≤50 vs. 51–65: ^a^: *p* < 0.05; ^b^: *p* < 0.01; ^c^: *p* < 0.001. Within phenotype group, ≤50 vs. >65: ^e^: *p* < 0.01; ^f^: *p* < 0.001. Within phenotype group, 51–65 vs. >65: ^g^: *p* < 0.05; ^h^: *p* < 0.01; ^i^: *p* < 0.001.

## Data Availability

Publicly available datasets were analyzed in this study. This data can be found here: https://www.hcup-us.ahrq.gov/db/hcupdatapartners.jsp (accessed on 28 January 2021).

## Supplementary Materials

The following are available online at https://www.mdpi.com/2072-6643/13/2/659/s1, Figure S1: CONSORT statement for patient flow chart, Figure S2: Race/ethnicity proportions among a random 2% inpatient sample of the general medicine population (GMP) and inpatients with cirrhosis from the National Inpatient Sample database stratified by age group (≤50, 51–64, and >65 years old) quantified cumulatively from data in the years 2010–2014, Figure S3: Race/ethnicity proportions among a random 2% inpatient sample of the general medicine population (GMP) with muscle loss phenotype and inpatients with cirrhosis with muscle loss phenotype from the National Inpatient Sample database stratified by age group (≤50, 51–64, and >65 years old) quantified cumulatively from data in the years 2010–2014, Figure S4: Race/ethnicity proportions among a random 2% inpatient sample of the general medicine population (GMP) with and without muscle loss phenotype from the National Inpatient Sample database stratified by age group (≤50, 51–64, and >65 years old) quantified cumulatively from data in the years 2010–2014, Figure S5: Race/ethnicity proportions among inpatients with cirrhosis with and without muscle loss phenotype from the National Inpatient Sample database stratified by age group (≤50, 51–64, and >65 years old) quantified cumulatively from data in the years 2010–2014. Table S1: Comorbidities, insurance type and geographic distribution of hospitalized general medical population and patients with cirrhosis stratified by age, Table S2: Comorbidities, insurance type and geographic distribution of hospitalized general medical population and patients with cirrhosis with muscle loss stratified by age, Table S3: Comorbidities, insurance type and geographic distribution of general medical population with and without muscle loss phenotype stratified by age, Table S4: Comorbidities, insurance type and geographic distribution of hospitalized patients with cirrhosis with and without muscle loss phenotype stratified by age, Table S5: Linear regression analysis for hospitalized general medicine population and patients with cirrhosis predicting variables associated with the Elixhauser comorbidity score, Table S6: Multivariate logistic regression of in-hospital mortality in age groups (≤50, 51–65, >65 years old) of hospitalized patients with cirrhosis, Table S7: Multivariate linear regression analysis predicting variables associated with increased length of stay in age groups (≤50, 51–65, >65 years old) of inpatients with cirrhosis, Table S8: Multivariate linear regression predicting variables associated with log transformed cost of hospitalization in age groups (≤50, 51–65, >65 years old) of cirrhotics patients.

## References

[1] Ortman J.M., Velkoff V.A., Hogan H. (2014). An Aging Nation: The Older Population in the United States.

[2] Kim D., Li A.A., Perumpail B.J., Gadiparthi C., Kim W., Cholankeril G., Glenn J.S., Harrison S.A., Younossi Z.M., Ahmed A. (2019). Changing Trends in Etiology-Based and Ethnicity-Based Annual Mortality Rates of Cirrhosis and Hepatocellular Carcinoma in the United States. Hepatology.

[3] Antunes A.C., Araújo D.A., Veríssimo M.T., Amaral T.F. (2017). Sarcopenia and hospitalisation costs in older adults: A cross-sectional study. Nutr. Diet..

[4] Hazra N.C., Rudisill C., Gulliford M.C. (2018). Determinants of health care costs in the senior elderly: Age, comorbidity, impairment, or proximity to death?. Eur. J. Health Econ..

[5] van Vugt J.L.A., Buettner S., Alferink L.J.M., Bossche N., de Bruin R.W.F., Darwish Murad S., Polak W.G., Metselaar H.J., IJzermans J.N.M. (2018). Low skeletal muscle mass is associated with increased hospital costs in patients with cirrhosis listed for liver transplantation-a retrospective study. Transpl. Int..

[6] Collaborators GBDC (2020). The global, regional, and national burden of cirrhosis by cause in 195 countries and territories, 1990–2017: A systematic analysis for the Global Burden of Disease Study 2017. Lancet Gastroenterol. Hepatol..

[7] Kamimura K., Sakamaki A., Kamimura H., Setsu T., Yokoo T., Takamura M., Terai S. (2019). Considerations of elderly factors to manage the complication of liver cirrhosis in elderly patients. World J. Gastroenterol..

[8] Durand F., Levitsky J., Cauchy F., Gilgenkrantz H., Soubrane O., Francoz C. (2019). Age and liver transplantation. J. Hepatol..

[9] Kim I.H., Kisseleva T., Brenner D.A. (2015). Aging and liver disease. Curr. Opin. Gastroenterol..

[10] Mooney H., Roberts R., Cooksley W.G., Halliday J.W., Powell L.W. (1985). Alterations in the liver with ageing. Clin. Gastroenterol..

[11] Premoli A., Paschetta E., Hvalryg M., Spandre M., Bo S., Durazzo M. (2009). Characteristics of liver diseases in the elderly: A review. Minerva Gastroenterol. Dietol..

[12] Dasarathy S. (2016). Cause and management of muscle wasting in chronic liver disease. Curr. Opin. Gastroenterol..

[13] Dasarathy S. (2013). Posttransplant Sarcopenia: An Underrecognized Early Consequence of Liver Transplantation. Dig. Dis. Sci..

[14] Dasarathy S. (2012). Consilience in sarcopenia of cirrhosis. J. Cachexia Sarcopenia Muscle.

[15] Periyalwar P., Dasarathy S. (2012). Malnutrition in Cirrhosis: Contribution and Consequences of Sarcopenia on Metabolic and Clinical Responses. Clin. Liver Dis..

[16] Vural A., Attaway A., Welch N., Zein J., Dasarathy S. (2020). Skeletal muscle loss phenotype in cirrhosis: A nationwide analysis of hospitalized patients. Clin. Nutr..

[17] Cruz-Jentoft A.J., Baeyens J.P., Bauer J.M., Boirie Y., Cederholm T., Landi F., Martin F.C., Michel J.P., Rolland Y., Schneider S.M. (2010). Sarcopenia: European consensus on definition and diagnosis: Report of the European Working Group on Sarcopenia in Older People. Age Ageing.

[18] Welch C., Hassan-Smith Z.K., Greig C.A., Lord J.M., Jackson T.A. (2018). Acute Sarcopenia Secondary to Hospitalisation—An Emerging Condition Affecting Older Adults. Aging Dis..

[19] English K.L., Paddon-Jones D. (2010). Protecting muscle mass and function in older adults during bed rest. Curr. Opin. Clin. Nutr. Metab. Care.

[20] Kortebein P., Ferrando A., Lombeida J., Wolfe R., Evans W.J. (2007). Effect of 10 Days of Bed Rest on Skeletal Muscle in Healthy Older Adults. JAMA.

[21] U.S. Department of Health and Human Services (1997). International Classification of Diseases, Clinical Modifications (ICD-9-CM).

[22] Fox K.M., Brooks J.M., Gandra S.R., Markus R., Chiou C.F. (2009). Estimation of Cachexia among Cancer Patients Based on Four Definitions. J. Oncol..

[23] Attaway A.H., Welch N., Hatipoğlu U., Zein J.G., Dasarathy S. (2021). Muscle loss contributes to higher morbidity and mortality in COPD: An analysis of national trends. Respirology.

[24] Covinsky K.E., Palmer R.M., Fortinsky R.H., Counsell S.R., Stewart A.L., Rn D.K., Ma C.J.B., Landefeld C.S. (2003). Loss of Independence in Activities of Daily Living in Older Adults Hospitalized with Medical Illnesses: Increased Vulnerability with Age. J. Am. Geriatr. Soc..

[25] Sam J., Nguyen G.C. (2009). Protein-calorie malnutrition as a prognostic indicator of mortality among patients hospitalized with cirrhosis and portal hypertension. Liver Int..

[26] Menendez M.E., Neuhaus V., Van Dijk N.C., Ring D. (2014). The Elixhauser Comorbidity Method Outperforms the Charlson Index in Predicting Inpatient Death After Orthopaedic Surgery. Clin. Orthop. Relat. Res..

[27] Little J., Higgins J.P., Ioannidis J.P., Moher D., Gagnon F., Von Elm E., Khoury M.J., Cohen B., Davey-Smith G., Grimshaw J.M. (2009). STrengthening the REporting of Genetic Association Studies (STREGA): An Extension of the STROBE Statement. Ann. Intern. Med..

[28] Bell B.P., Manos M.M., Zaman A., Terrault N., Thomas A., Navarro V.J., Dhotre K.B., Murphy R.C., Van Ness G.R., Stabach N. (2008). The Epidemiology of Newly Diagnosed Chronic Liver Disease in Gastroenterology Practices in the United States: Results from Population-Based Surveillance. Am. J. Gastroenterol..

[29] Orman E.S., Roberts A., Ghabril M., Nephew L., Desai A.P., Patidar K., Chalasani N. (2019). Trends in Characteristics, Mortality, and Other Outcomes of Patients with Newly Diagnosed Cirrhosis. JAMA Netw. Open.

[30] Aduen J.F., Sujay B., Dickson R.C., Heckman M.G., Hewitt W.R., Stapelfeldt W.H., Steers J.L., Harnois D.M., Kramer D.J. (2009). Outcomes after liver transplant in patients aged 70 years or older compared with those younger than 60 years. Mayo Clin. Proc..

[31] Parrish N.F., Feurer I.D., Matsuoka L.K., Rega S.A., Perri R., Alexopoulos S.P. (2019). The Changing Face of Liver Transplantation in the United States: The Effect of HCV Antiviral Eras on Transplantation Trends and Outcomes. Transplant. Direct.

[32] Su F., Yu L., Berry K., Liou I.W., Landis C.S., Rayhill S.C., Reyes J.D., Ioannou G.N. (2016). Aging of Liver Transplant Registrants and Recipients: Trends and Impact on Waitlist Outcomes, Post-Transplantation Outcomes, and Transplant-Related Survival Benefit. Gastroenterology.

[33] Wong R.J., Singal A.K. (2020). Trends in Liver Disease Etiology Among Adults Awaiting Liver Transplantation in the United States, 2014–2019. JAMA Netw. Open.

[34] Haugen C.E., McAdams-DeMarco M., Holscher C.M., Ying H., Gurakar A.O., Garonzik-Wang J., Cameron A.M., Segev D.L., Lai J.C. (2020). Multicenter Study of Age, Frailty, and Waitlist Mortality Among Liver Transplant Candidates. Ann. Surg..

[35] Sharpton S.R., Feng S., Hameed B., Yao F., Lai J.C. (2014). Combined effects of recipient age and model for end-stage liver disease score on liver transplantation outcomes. Transplantation.

[36] Van Wagner L.B., Harinstein M.E., Runo J.R., Darling C., Serper M., Hall S., Kobashigawa J.A., Hammel L.L. (2018). Multidisciplinary approach to cardiac and pulmonary vascular disease risk assessment in liver transplantation: An evaluation of the evidence and consensus recommendations. Am. J. Transplant..

[37] Watt K.D., Pedersen R.A., Kremers W.K., Heimbach J.K., Charlton M.R. (2010). Evolution of Causes and Risk Factors for Mortality Post-Liver Transplant: Results of the NIDDK Long-Term Follow-Up Study. Arab. Archaeol. Epigr..

[38] Dellon E.S., Galanko J.A., Medapalli R.K., Russo M.W. (2006). Impact of Dialysis and Older Age on Survival after Liver Transplantation. Am. J. Transplant..

[39] Wang C.W., Feng S., Covinsky K.E., Hayssen H., Zhou L.Q., Yeh B.M., Lai J.C. (2016). A Comparison of Muscle Function, Mass, and Quality in Liver Transplant Candidates: Results from the Functional Assessment in Liver Transplantation Study. Transplantation.

[40] Tapper E.B., Finkelstein D., Mittleman M.A., Piatkowski G., Lai M. (2015). Standard assessments of frailty are validated predictors of mortality in hospitalized patients with cirrhosis. Hepatology.

[41] Marineo G., Marotta F., Sisti G. (2004). Cirrhosis Progression as a Model of Accelerated Senescence: Affecting the Biological Aging Clock by a Breakthrough Biophysical Methodology. Ann. N. Y. Acad. Sci..

[42] Aravinthan A.D., Alexander G.J. (2016). Senescence in chronic liver disease: Is the future in aging?. J. Hepatol..

[43] McDaniel J.G., Davuluri G., Hill E.A., Moyer M., Runkana A., Prayson R., Van Lunteren E., Dasarathy S. (2016). Hyperammonemia results in reduced muscle function independent of muscle mass. Am. J. Physiol. Gastrointest. Liver Physiol..

[44] Davuluri G., Allawy A., Thapaliya S., Rennison J.H., Singh D., Kumar A., Sandlers Y., Van Wagoner D.R., Flask C.A., Hoppel C. (2016). Hyperammonaemia-induced skeletal muscle mitochondrial dysfunction results in cataplerosis and oxidative stress. J. Physiol..

[45] Salim T.I., Nestlerode L.C., Lucatorto E.L., Wasserman T.L., Din H.A., Landsittel D.P., Tevar A.D., Johnson J.T., Duarte-Rojo A., Dunn M.A. (2020). Frailty as Tested by Gait Speed Is a Risk Factor for Liver Transplant Respiratory Complications. Am. J. Gastroenterol..

[46] Huisman E.J., Trip E.J., Siersema P.D., Van Hoek B., Van Erpecum K.J. (2011). Protein energy malnutrition predicts complications in liver cirrhosis. Eur. J. Gastroenterol. Hepatol..

[47] Vaz J., Eriksson B., Strömberg U., Buchebner D., Midlöv P. (2020). Incidence, aetiology and related comorbidities of cirrhosis: A Swedish population-based cohort study. BMC Gastroenterol..

[48] Mukthinuthalapati V.V.P.K., Akinyeye S., Fricker Z.P., Syed M., Orman E.S., Nephew L., Vilar-Gomez E., Slaven J., Chalasani N., Balakrishnan M. (2019). Early predictors of outcomes of hospitalization for cirrhosis and assessment of the impact of race and ethnicity at safety-net hospitals. PLoS ONE.

[49] Tsien C., Garber A., Narayanan A., Shah S.N., Barnes D., Eghtesad B., Fung J., McCullough A.J., Dasarathy S. (2014). Post-liver transplantation sarcopenia in cirrhosis: A prospective evaluation. J. Gastroenterol. Hepatol..

[50] Pagadala M., Dasarathy S., Eghtesad B., McCullough A.J. (2009). Posttransplant metabolic syndrome: An epidemic waiting to happen. Liver Transplant..

[51] Dominguez L.J., Barbagallo M. (2016). The biology of the metabolic syndrome and aging. Curr. Opin. Clin. Nutr. Metab. Care.

[52] Guirguis J., Chhatwal J., Dasarathy J., Rivas J.M., McMichael D., Nagy L.E., McCullough A.J., Dasarathy S. (2015). Clinical Impact of Alcohol-Related Cirrhosis in the Next Decade: Estimates Based on Current Epidemiological Trends in the United States. Alcohol. Clin. Exp. Res..

[53] Sajja K.C., Mohan D.P., Rockey D.C. (2014). Age and Ethnicity in Cirrhosis. J. Investig. Med..

[54] Bond M.J., Herman A.A. (2016). Lagging Life Expectancy for Black Men: A Public Health Imperative. Am. J. Public Health.

