# Supplementary material for: Compound Sarcopenia in Hospitalized Patients with Cirrhosis Worsens Outcomes with Increasing Age
Source: Nutrients. 2021 Feb 18;13(2):659. doi: 10.3390/nu13020659 (PMC7923160; doi:10.3390/nu13020659)
Supplement: Supplementary file 1 [file nutrients-13-00659-s001.pdf]

Supplementary Figure legends.

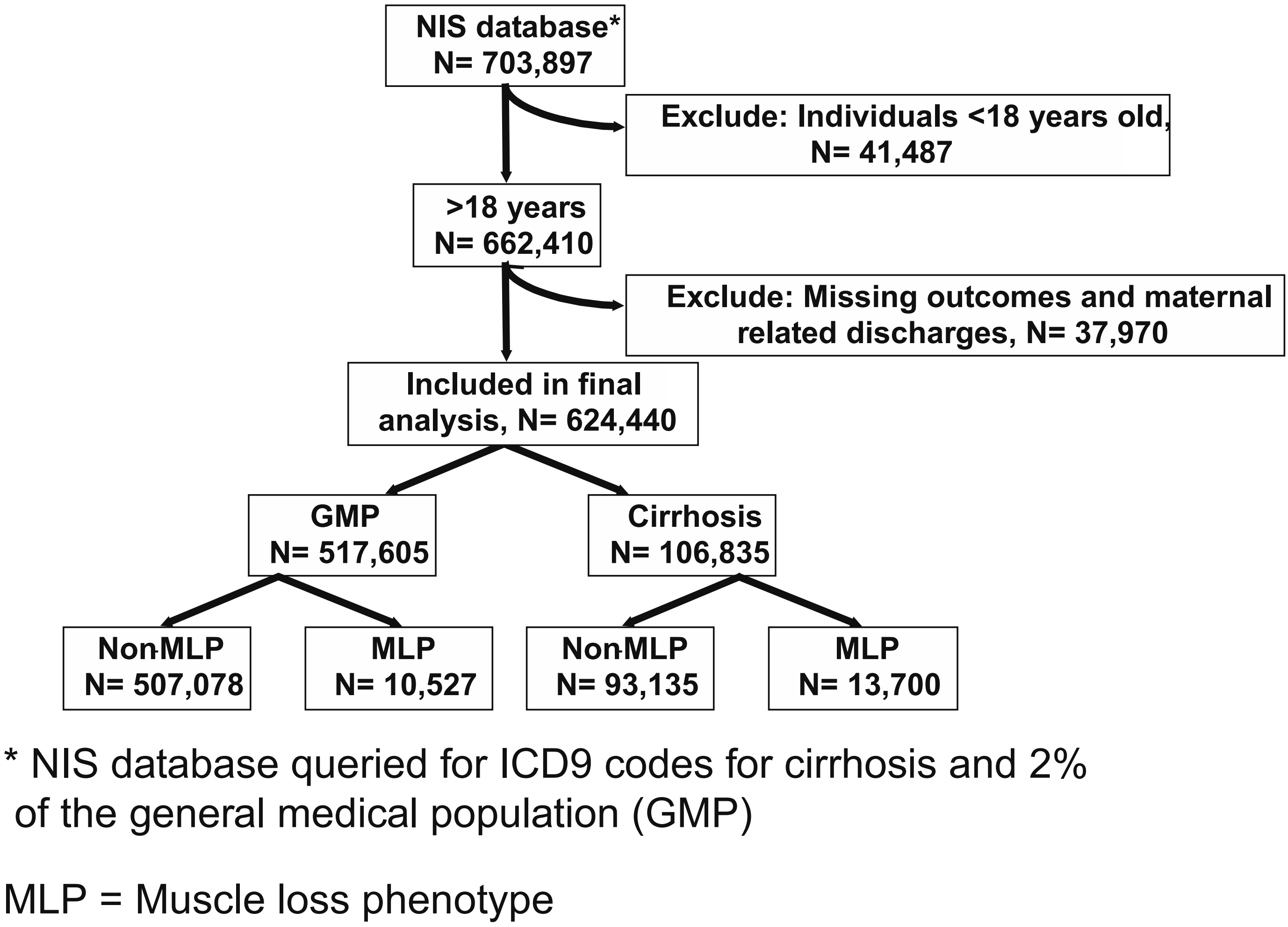

Supplementary Figure S1. CONSORT statement for patient flow chart.

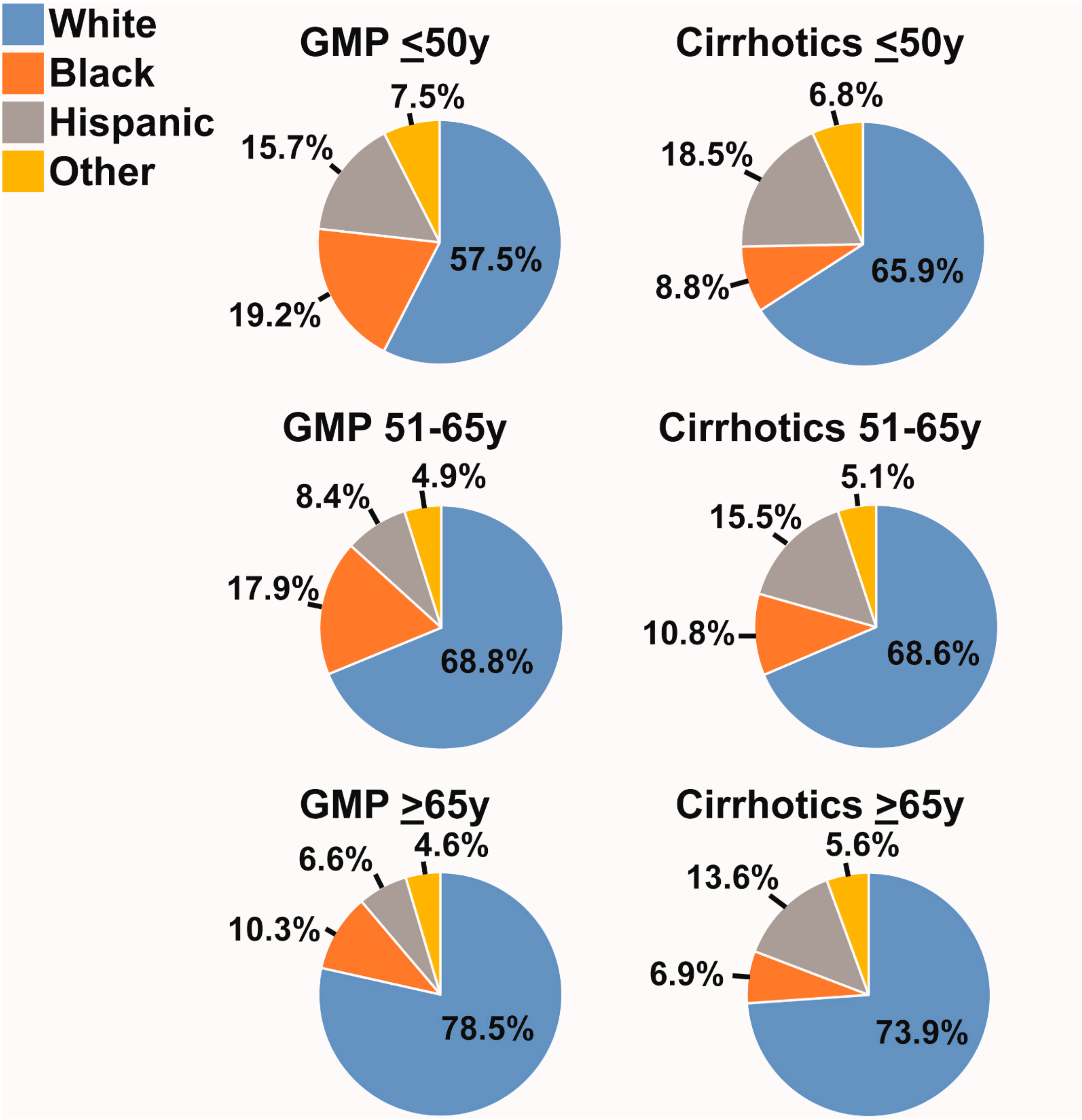

Supplementary Figure S2. Race/ethnicity proportions among a random 2% inpatient sample of the general medicine population (GMP) and inpatient cirrhotics from the National Inpatient Sample database stratified by age group (50 years(y), 51-64y, and >65y of age) quantified cumulatively from data in the years 2010-2014.

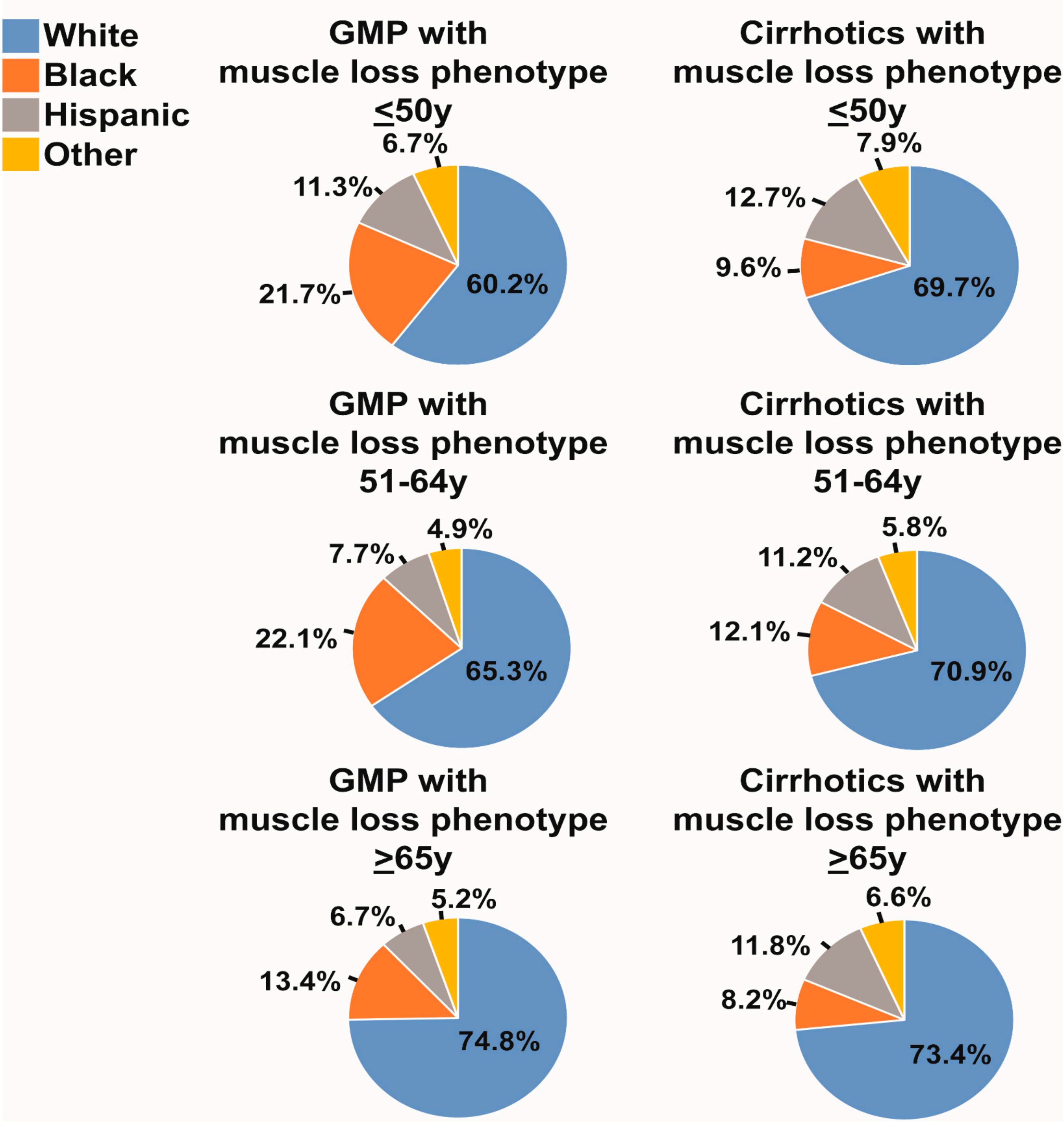

Supplementary Figure S3. Race/ethnicity proportions among a random 2% inpatient sample of the general medicine population (GMP) with muscle loss phenotype and inpatient cirrhotics with muscle loss phenotype from the National Inpatient Sample database stratified by age group (50 years(y), 51-64y, and >65y of age) quantified cumulatively from data in the years 2010-2014.

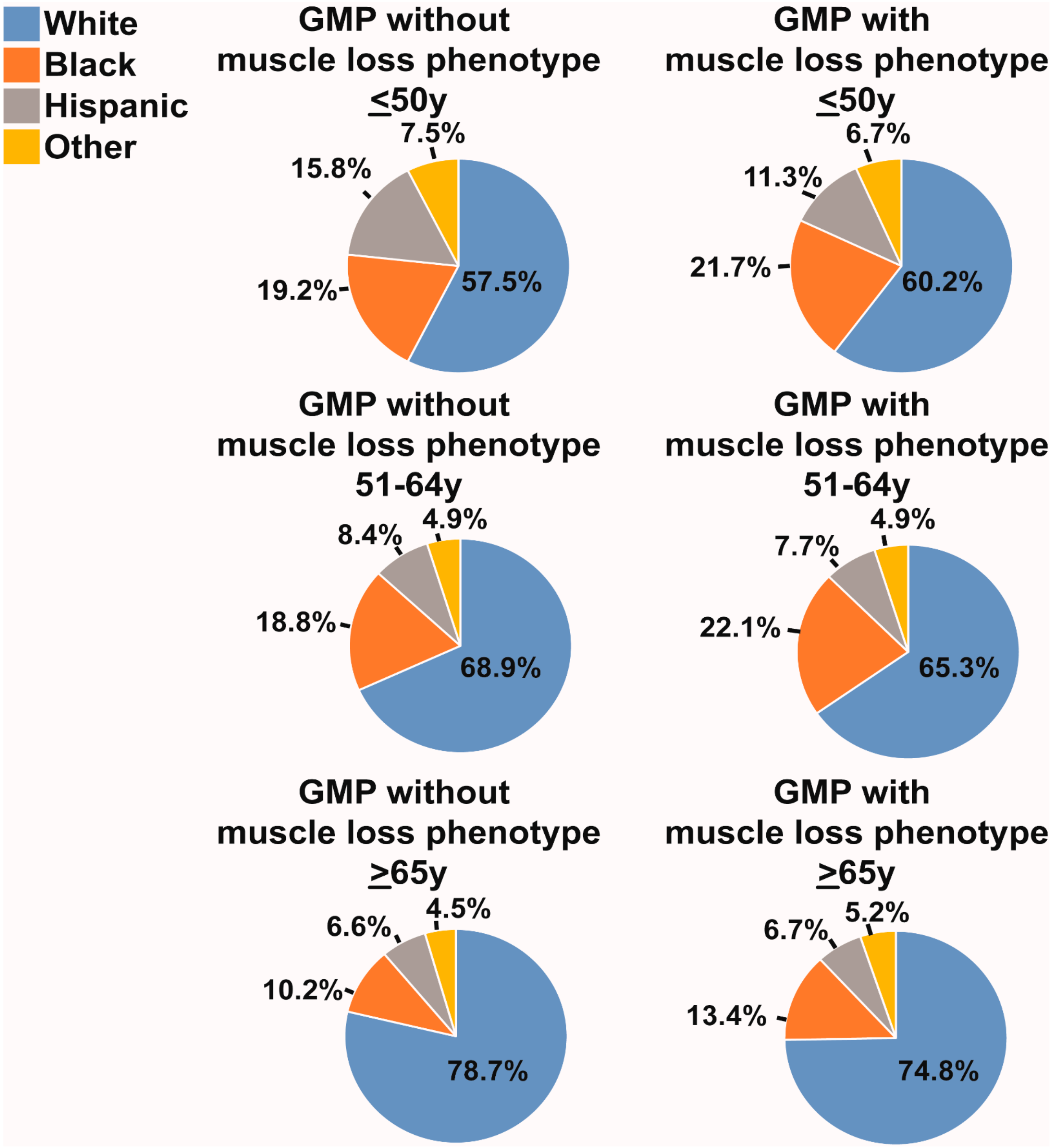

Supplementary Figure S4. Race/ethnicity proportions among a random 2% inpatient sample of the general medicine population (GMP) with and without muscle loss phenotype from the National Inpatient Sample database stratified by age group (50 years(y),_51-64y, and ≥65y of age) quantified cumulatively from data in the years 2010-2014.

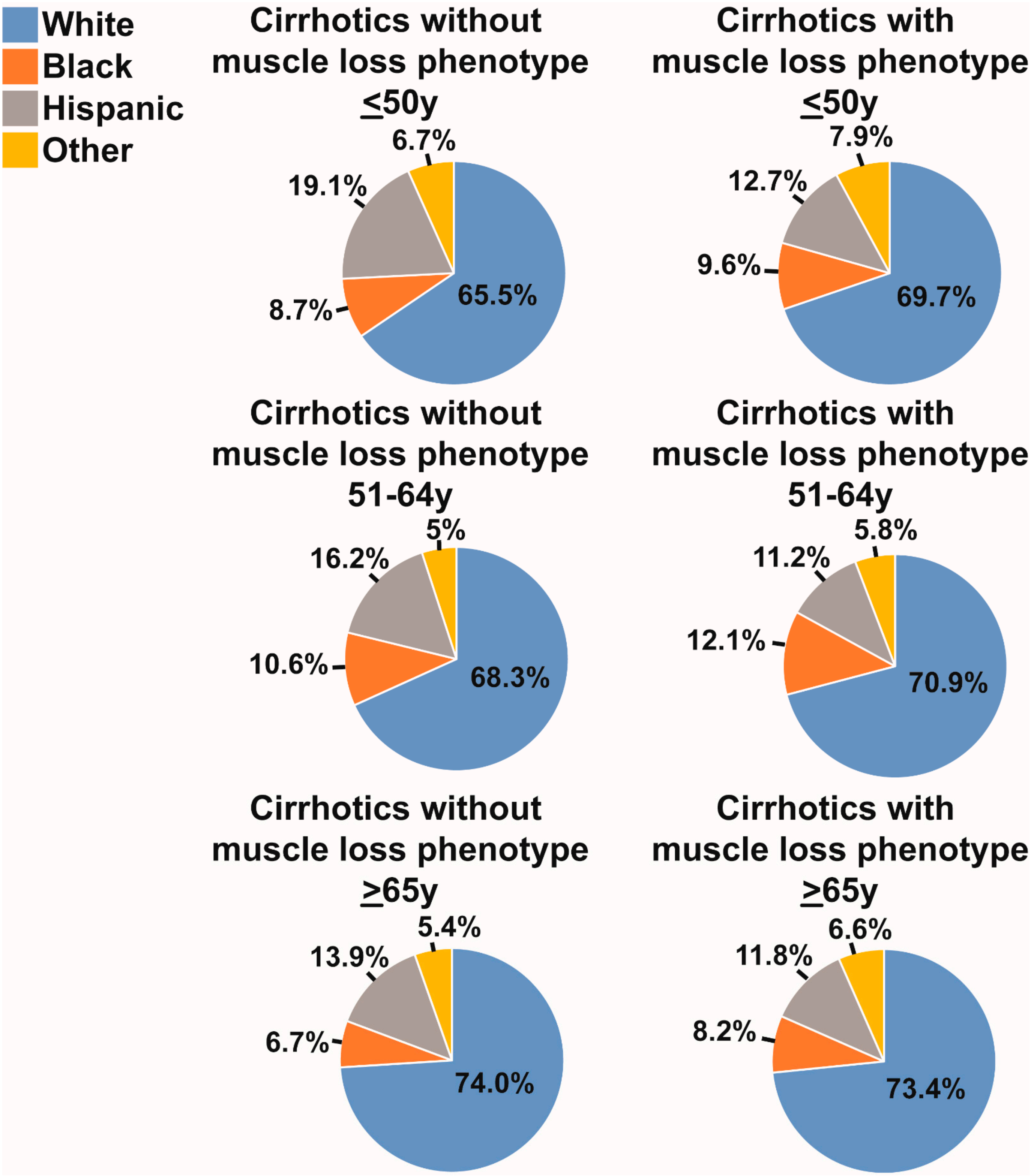

Supplementary Figure S5. Race/ethnicity proportions among inpatient cirrhotics with and without muscle loss phenotype from the National Inpatient Sample database stratified by age group (50 years(y), 51-64y, and ≥65y of age) quantified cumulatively from data in the years 2010-2014.

Supplementary Table S1. Comorbidities, insurance type and geographic distribution of hospitalized general medical and Cirrhosis patients stratified by age.

	GMP			*P* value [a]	Cirrhotics			*P* value [a]
Age categories (years)	≤50	51-65	>65		≤50	51-65	>65	
Number of patients	188,510	121,829	207,266		24,848	52,969	29,018	
Comorbidities (%)								
Anemia	8,476 (4.5)	1,174 (1.0)	3,061 (1.5)	<0.001	1,293 (5.2)***	2,461 (4.6)***	1,399 (4.8)***	0.003
Coagulopathy	5,464 (2.9)	6,398 (5.3)	11,583 (5.6)	<0.001	12,439 (50.1)***	24,618 (46.5)***	11,289 (38.9)***	<0.001
Congestive heart failure	2,775 (1.5)	8,555 (7.0)	31,797 (15.3)	<0.001	1,567 (6.3)***	6,831 (12.9)***	8,583 (29.6)***	<0.001
Depression	15,258 (8.1)	16,431 (13.5)	22,887 (11.0)	<0.001	3,568 (14.4)***	7,794 (14.7)***	3,884 (13.4)***	<0.001
Drug abuse	14,559 (7.7)	5,546 (4.6)	1,082 (0.5)	<0.001	3,157 (12.7)***	5,341 (10.1)***	511 (1.8)***	<0.001
Hypothyroidism	8,246 (4.4)	12,761 (10.5)	36,382 (17.6)	<0.001	1,774 (7.1)***	5,813 (11.0)	6,449 (22.2)***	<0.001
Lymphoma	518 (0.3)	912 (0.7)	2,581 (1.2)	<0.001	89 (0.4)	347 (0.7)	432 (1.5)	<0.001
Metastatic cancer	1,525 (0.8)	3,793 (3.1)	5,854 (2.8)	<0.001	148 (0.6)	889 (1.7)***	868 (3.0)	<0.001
Peptic ulcer disease	25 (0.0)	43 (0.0)	79 (0.0)	<0.001	17 (0.1)	57 (0.1)	29 (0.1)	0.253
Solid tumors	993 (0.5)	2,869 (2.4)	6,319 (3.0)	<0.001	449 (1.8)***	2,781 (5.3)***	1,718 (5.9)***	<0.001
Disposition of patient upon discharge (%)[†]				<0.001				<0.001
Transfer to short-term hospital	2,532 (1.4)	3,115 (2.6)	5,069 (2.5)		887 (3.9)***	1,881 (3.9)***	848 (3.3)***	
Transfer other: SNF, ICF	6,657 (3.6)	13,835 (11.6)	61,557 (30.9)		2,268 (9.9)***	8,620 (18.0)***	8,810 (34.4)***	
Home Health Care	8,503 (4.5)	16,678 (14.0)	39,136 (19.7)		2,096 (9.2)***	6,565 (13.7)	5,527 (21.6)***	
Against medical advice	3,580 (1.9)	1,679 (1.4)	860 (0.4)		838 (3.7)***	1,019 (2.1)***	159 (0.6)	
Urban-rural classification for US county (%)				<0.001				<0.001
Metro areas ≥ 1 million	60,556 (32.1)	36,042 (29.6)	55,065 (26.6)		8,377 (33.7)	18,148 (34.3)	9,439 (32.5)	

Metro areas of 250,000-999,999	47,470 (25.2)	30,118 (24.7)	52,678 (25.4)		4,902 (19.7) ***	9,856 (18.6) ***	5,104 (17.6) ***	
Metro areas of 50,000-249,999	34,824 (18.5)	22,343 (18.3)	37,917 (18.3)		2,079 (8.4) ***	4,712 (8.9) ***	2,534 (8.7) ***	
Fringe counties of metro ≥ 1 million	16,693 (8.9)	11,349 (9.3)	19,860 (9.6)		5,634 (22.7)	11,850 (22.4)	6,977 (24.0)	
Micropolitan counties	17,426 (9.2)	12,753 (10.5)	23,711 (11.4)		2,313 (9.3)	5,057 (9.5)	2,899 (10.0)	
Non-metro or micropolitan counties	11,541 (6.1)	9,224 (7.6)	18,035 (8.7)		1,543 (6.2)	3,346 (6.3)	2,065 (7.1)	
Mean household income by zip code (%)				<0.001				<0.001
0-25th percentile	59,511 (31.6)	38,741 (31.8)	57,957 (28.0)		9,190 (37.0)	19,693 (37.2)	8,955 (30.9)	
26th to 50th percentile	47,139 (25.0)	31,305 (25.7)	54,145 (26.1)		6,369 (25.6)	13,959 (26.4)	7,641 (26.3)	
51st to 75th percentile	44,382 (23.5)	28,037 (23.0)	49,625 (23.9)		5,546 (22.3)	11,549 (21.8)	6,796 (23.4)	
76th to 100th percentile	37,478 (19.9)	23,746 (19.5)	45,539 (22.0)		3,743 (15.1)	7,768 (14.7)	5,626 (19.4)	
Primary payer for insurance (%)				<0.001				<0.001
Medicare	16,905 (9.0)	35,297 (29.0)	185,937 (89.7)		3,711 (14.9)	15,660 (29.6)	25,343 (87.3)	
Medicaid	59,866 (31.8)	18,110 (14.9)	2,983 (1.4)		9,680 (39.0)	14,411 (27.2)	709 (2.4)	
Private insurance	80,778 (42.9)	53,126 (43.6)	15,231 (7.3)		452 (1.8)	596 (1.1)	16 (0.1)	
Self-pay	19,678 (10.4)	8,349 (6.9)	945 (0.5)		1,422 (5.7)	2,685 (5.1)	412 (1.4)	
No pay	2,011 (1.1)	1,041 (0.9)	122 (0.1)		5,352 (21.5)	14,143 (26.7)	2,314 (8.0)	
Other	9,272 (4.9)	5,906 (4.8)	2,048 (1.0)		4,231 (17.0)	5,474 (10.3)	224 (0.8)	

[†]Routine discharge is included in the total percentage and is shown in the main tables

Abbreviations: GMP: General medical population, ICF: Intermediate care facility, SNF: Skilled nursing facility.

[a] p values that are marked represent ANOVA analyses.

GMP without muscle loss vs. cirrhotics without muscle loss, between each age group: * p <0.05, ** p <0.01, *** p <0.001.

Supplementary Table S2. Comorbidities, insurance type and geographic distribution of hospitalized general medical and cirrhosis patients with muscle loss stratified by age.

	GMP with muscle loss phenotype			*P* value [a]	Cirrhotics with muscle loss phenotype			*P* value [a]
Age categories (years)	≤50	51-65	>65		≤50	51-65	>65	
Number of patients	1,058	2,417	7,052		2,511	6,688	4,501	
Comorbidities (%)								
Anemia	33 (3.1)	71 (2.9)	197 (2.8)	0.811	135 (5.4) ***	300 (4.5) ***	205 (4.6) ***	0.177
Coagulopathy	230 (21.7)	510 (21.1)	1,070 (15.2)	<0.001	1,652 (65.8)	3,994 (59.7)	2,061 (45.8)	<0.001
Congestive heart failure	99 (9.4)	476 (19.7)	2,177 (30.9)	<0.001	240 (9.6)	1,099 (16.4) ***	1,518 (33.7) **	<0.001
Depression	207 (19.6)	453 (18.7)	1,065 (15.1)	<0.001	514 (20.5)	1,216 (18.2)	683 (15.2)	<0.001
Drug abuse	162 (15.3)	177 (7.3)	68 (1.0)	<0.001	413 (16.4)	857 (12.8) ***	117 (2.6) ***	<0.001
Hypothyroidism	92 (8.7)	333 (13.8)	1,493 (21.2)	<0.001	272 (10.8)	797 (11.9)	1,069 (23.8) **	<0.001
Lymphoma	18 (1.7)	42 (1.7)	166 (2.4)	0.113	17 (0.7) **	61 (0.9) **	83 (1.8) *	<0.001
Metastatic cancer	71 (6.7)	240 (9.9)	551 (7.8)	0.001	39 (1.6) ***	234 (3.5) ***	240 (5.3) ***	<0.001
Peptic ulcer disease	1 (0.1)	1 (0.0)	7 (0.1)	0.699	2 (0.1)	10 (0.1)	3 (0.1)	0.379
Solid tumors	18 (1.7)	130 (5.4)	392 (5.6)	<0.001	59 (2.3)	422 (6.3)	341 (7.6) ***	<0.001
Disposition of patient upon discharge (%)[†]				<0.001				<0.001
Transfer to short-term hospital	55 (5.6)	103 (4.8)	183 (2.9)		123 (6.0)	259 (4.8)	125 (3.5)	
Transfer other: SNF, ICF	285 (28.8)	887 (41.5)	3,683 (59.3)		565 (27.4)	2,152 (39.6)	1,940 (54.0) ***	
Home Health Care	241 (24.4)	526 (24.6)	1,420 (22.9)		387 (18.8) ***	1,099 (20.2)	826 (23.0)	
Against medical advice	14 (1.4)	12 (0.6)	16 (0.3)		42 (2.0)	82 (1.5) ***	22 (0.6)	
Urban-rural classification for US county (%)								

Metro areas ≥ 1 million	371 (35.1)	828 (34.3)	2,369 (33.6)	0.054	890 (35.4)	2387 (35.7)	1,633 (36.3) **	0.873
Metro areas of 250,000-999,999	233 (22.0)	564 (23.3)	1,844 (26.1)		464 (18.5) ***	1,201 (18.0) ***	803 (17.8) ***	
Metro areas of 50,000-249,999	167 (15.8)	407 (16.8)	1129 (16.0)		222 (8.8) ***	610 (9.1) ***	371 (8.2) ***	
Fringe counties of metro ≥ 1 million	100 (9.5)	239 (9.9)	611 (8.7)		541 (21.5) ***	1,453 (21.7) ***	1,005 (22.3) ***	
Micropolitan counties	114 (10.8)	220 (9.1)	641 (9.1)		228 (9.1)	630 (9.4)	404 (9.0)	
Non-metro or micropolitan counties	73 (6.9)	159 (6.6)	458 (6.5)		166 (6.6)	407 (6.1)	285 (6.3)	
Mean household income by zip code (%)				<0.001				<0.001
0-25th percentile	382 (36.1)	842 (34.8)	1,950 (27.7)		885 (35.2)	2,374 (35.5)	1,349 (30.0)	
26th to 50th percentile	269 (25.4)	634 (26.2)	1,703 (24.1)		619 (24.7)	1,779 (26.6)	1,157 (25.7)	
51st to 75th percentile	259 (24.5)	519 (21.5)	1,779 (25.2)		588 (23.4)	1,473 (22.0)	1,100 (24.4)	
76th to 100th percentile	148 (14.0)	422 (17.5)	1,620 (23.0)		419 (16.7)	1,062 (15.9)	895 (19.9) ***	
Primary payer for insurance (%)				<0.001				<0.001
Medicare	300 (28.4)	936 (38.7)	6,396 (90.7)		481 (19.2) ***	2,263 (33.8) ***	3,996 (88.8) ***	
Medicaid	319 (30.2)	492 (20.4)	94 (1.3)		1,010 (40.2) ***	1,776 (26.6) ***	96 (2.1) ***	
Private insurance	301 (28.4)	767 (31.7)	468 (6.6)		587 (23.4) ***	1,826 (27.3) ***	329 (7.3)	
Self-pay	82 (7.8)	131 (5.4)	30 (0.4)		282 (11.2) ***	491 (7.3) **	26 (0.6)	
No pay	14 (1.3)	12 (0.5)	4 (0.1)		36 (1.4)	39 (0.6)	3 (0.1)	
Other	42 (4.0)	79 (3.3)	60 (0.9)		115 (4.6)	293 (4.4) *	51 (1.1)	

[†]Routine discharge is included in the total percentage and is shown in the main tables
GMP: General medical population, ICF: Intermediate care facility, SNF: Skilled nursing facility.
[a] p values that are marked represent ANOVA analyses.
GMP with muscle loss vs cirrhotics with muscle loss, between each age group: * *p* <0.05, ** *p* <0.01, *** *p* <0.001.

Supplementary Table S3. Comorbidities, insurance type and geographic distribution of general medical patients with and without muscle loss phenotype stratified by age.

	GMP without muscle loss phenotype			*P* value [a]	GMP with muscle loss phenotype			*P* value [a]
Age categories (years)	≤50	51-65	>65		≤50	51-65	>65	
Number of patients	187,452	119,412	200,214		1,058	2,417	7,052	
Comorbidities (%)								
Anemia	8,443 (4.5)	1,103 (0.9)	2,864 (1.4)	<0.001	33 (3.1) [**]	71 (2.9) [***]	197 (2.8) [***]	0.811
Coagulopathy	5,234 (2.8)	5,888 (4.9)	10,513 (5.3)	<0.001	230 (21.7) [***]	510 (21.1) [***]	1,070 (15.2) [***]	<0.001
Congestive heart failure	2,676 (1.4)	8,079 (6.8)	29,620 (14.8)	<0.001	99 (9.4) [***]	476 (19.7) [***]	2,177 (30.9) [***]	<0.001
Depression	15,051 (8.0)	15,978 (13.4)	21,822 (10.9)	<0.001	207 (19.6) [***]	453 (18.7) [***]	1,065 (15.1) [***]	<0.001
Drug abuse	14,397 (7.7)	5,369 (4.5)	1014 (0.5)	<0.001	162 (15.3) [***]	177 (7.3) [***]	68 (1.0) [***]	<0.001
Hypothyroidism	8,154 (4.3)	12,428 (10.4)	34,889 (17.4)	<0.001	92 (8.7) [***]	333 (13.8) [***]	1,493 (21.2) [***]	<0.001
Lymphoma	500 (0.3)	870 (0.7)	2,415 (1.2)	<0.001	18 (1.7) [***]	42 (1.7) [***]	166 (2.4) [***]	0.113
Metastatic cancer	1,454 (0.8)	3,553 (3.0)	5,303 (2.6)	<0.001	71 (6.7) [***]	240 (9.9) [***]	551 (7.8) [***]	0.001
Peptic ulcer disease	24 (0.0)	42 (0.0)	72 (0.0)	<0.001	1 (0.1)	1 (0.0)	7 (0.1)	0.699
Solid tumors	975 (0.5)	2,739 (2.3)	5,927 (3.0)	<0.001	18 (1.7) [***]	130 (5.4) [***]	392 (5.6) [***]	<0.001
Disposition of patient upon discharge (%)[†]				<0.001				<0.001
Transfer to short-term hospital	2,477 (1.3)	3,012 (2.6)	4,886 (2.5)		55 (5.6) [***]	103 (4.8) [***]	183 (2.9)	
Transfer other: SNF, ICF	6,372 (3.4)	12,948 (11.0)	57,874 (30.0)		285 (28.8) [***]	887 (41.5) [***]	3,683 (59.3) [***]	
Home Health Care	8,262 (4.4)	16,152 (13.8)	37,716 (19.6)		241 (24.4) [***]	526 (24.6) [***]	1,420 (22.9) [***]	
Against medical advice	3,566 (1.9)	1,667 (1.4)	844 (0.4)		14 (1.4)	12 (0.6) [***]	16 (0.3)	
Urban-rural classification for US county (%)				<0.001				0.054
Metro areas ≥ 1 million	60,185 (32.1)	35,214 (29.5)	52,696 (26.3)		371 (35.1) [*]	828 (34.3) [***]	2,369 (33.6) [***]	

Metro areas of 250,000-999,999	47,237 (25.2)	29,554 (24.7)	50,834 (25.4)		233 (22.0) *	564 (23.3)	1,844 (26.1)	
Metro areas of 50,000-249,999	34,657 (18.5)	21,936 (18.4)	36,788 (18.4)		167 (15.8) *	407 (16.8) *	1,129 (16.0) **	
Fringe counties of metro ≥ 1 million	16,593 (8.9)	11,110 (9.3)	19,249 (9.6)		100 (9.5)	239 (9.9)	611 (8.7)	
Micropolitan counties	17,312 (9.2)	12,533 (10.5)	23,070 (11.5)		114 (10.8)	220 (9.1) *	641 (9.1) ***	
Non-metro or micropolitan counties	11,468 (6.1)	9,065 (7.6)	17,577 (8.8)		73 (6.9)	159 (6.6)	458 (6.5) ***	
Mean household income by zip code (%)								
0-25th percentile	59,129 (31.5)	37,899 (31.7)	56,007 (28.0)		382 (36.1) **	842 (34.8) **	1,950 (27.7)	
26th to 50th percentile	46,870 (25.0)	30,671 (25.7)	52,442 (26.2)	<0.001	269 (25.4)	634 (26.2)	1,703 (24.1) *	<0.001
51st to 75th percentile	44,123 (23.5)	27,518 (23.0)	47,846 (23.9)		259 (24.5)	519 (21.5)	1,779 (25.2)	
76th to 100th percentile	37,330 (19.9)	23,324 (19.5)	43,919 (21.9)		148 (14.0) ***	422 (17.5) **	1,620 (23.0)	
Primary payer for insurance (%)								
Medicare	16,605 (8.9)	34,361 (28.8)	179,541 (89.7)		300 (28.4) ***	936 (38.7) ***	6,396 (90.7) **	
Medicaid	59,547 (31.8)	17,618 (14.8)	2889 (1.4)		319 (30.2)	492 (20.4) ***	94 (1.3)	
Private insurance	80,477 (42.9)	52,359 (43.8)	14,763 (7.4)	<0.001	301 (28.4) ***	767 (31.7) ***	468 (6.6)	<0.001
Self-pay	19,596 (10.5)	8218 (6.9)	915 (0.5)		82 (7.8) **	131 (5.4) **	30 (0.4)	
No pay	1,997 (1.1)	1,029 (0.9)	118 (0.1)		14 (1.3)	12 (0.5) *	4 (0.1)	
Other	9,230 (4.9)	5,827 (4.9)	1,988 (1.0)		42 (4.0)	79 (3.3) ***	60 (0.9)	

[†]Routine discharge is included in the total percentage and is shown in the main tables

Abbreviations: GMP: General medical population, ICF: Intermediate care facility, SNF: Skilled nursing facility.

[a]*p* values that are marked represent ANOVA analyses. GMP without muscle loss vs GMP with muscle loss, between each age group: * $p < 0.05$, ** $p < 0.01$, *** $p < 0.001$.

Supplementary Table S4. Comorbidities, insurance type and geographic distribution of hospitalized cirrhosis patients with and without muscle loss phenotype stratified by age.

	Cirrhotics without muscle loss phenotype			*P* value [a]	Cirrhotics with muscle loss phenotype			*P* value [a]
Age categories (years)	≤50	51-65	>65		≤50	51-65	>65	
Number of patients	22,337	46,281	24,517		2,511	6,688	4,501	
Comorbidities (%)								
Anemia	1,158 (5.2)	2,161 (4.7)	1,194 (4.9)	0.013	135 (5.4)	300 (4.5)	205 (4.6)	0.177
Coagulopathy	10,787 (48.3)	20,624 (44.6)	9,228 (37.6)	<0.001	1,652 (65.8) ***	3,994 (59.7) ***	2,061 (45.8) ***	<0.001
Congestive heart failure	1,327 (5.9)	5,732 (12.4)	7,065 (28.8)	<0.001	240 (9.6) ***	1,099 (16.4) ***	1,518 (33.7) ***	<0.001
Depression	3,054 (13.7)	6,578 (14.2)	3,201 (13.1)	<0.001	514 (20.5) ***	1,216 (18.2) ***	683 (15.2) ***	<0.001
Drug abuse	2,744 (12.3)	4,484 (9.7)	394 (1.6)	<0.001	413 (16.4) ***	857 (12.8) ***	117 (2.6) ***	<0.001
Hypothyroidism	1,502 (6.7)	5,016 (10.8)	5,380 (21.9)	<0.001	272 (10.8) ***	797 (11.9) **	1,069 (23.8) **	<0.001
Lymphoma	72 (0.3)	286 (0.6)	349 (1.4)	<0.001	17 (0.7) **	61 (0.9) **	83 (1.8) *	<0.001
Metastatic cancer	109 (0.5)	655 (1.4)	628 (2.6)	<0.001	39 (1.6) ***	234 (3.5) ***	240 (5.3) ***	<0.001
Peptic ulcer disease	15 (0.1)	47 (0.1)	26 (0.1)	0.307	2 (0.1)	10 (0.1)	3 (0.1)	0.379
Solid tumors	390 (1.7)	2,359 (5.1)	1,377 (5.6)	<0.001	59 (2.3) *	422 (6.3) ***	341 (7.6) ***	<0.001
Disposition of patient upon discharge (%)[†]				<0.001				<0.001
Transfer to short-term hospital	764 (3.7)	1622 (3.8)	723 (3.3)		123 (6.0) *	259 (4.8) ***	125 (3.5)	
Transfer other: SNF, ICF	1,703 (8.2)	6,468 (15.3)	6,870 (31.2)		565 (27.4) ***	2,152 (39.6) ***	1,940 (54.0) ***	
Home Health Care	1,709 (8.2)	5,466 (12.9)	4,701 (21.4)		387 (18.8) ***	1,099 (20.2) ***	826 (23.0) ***	
Against medical advice	796 (3.8)	937 (2.2)	137 (0.6)		42 (2.0) ***	82 (1.5) ***	22 (0.6)	
Urban-rural classification for US county (%)								

Metro areas ≥ 1 million	7,487 (33.5)	15,761 (34.1)	7,806 (31.8)	<0.001	890 (35.4)	2,387 (35.7) *	1,633 (36.3) ***	0.873
Metro areas of 250,000-999,999	4,438 (19.9)	8,655 (18.7)	4,301 (17.5)		464 (18.5)	1,201 (18.0)	803 (17.8)	
Metro areas of 50,000-249,999	1,857 (8.3)	4,102 (8.9)	2,163 (8.8)		222 (8.8)	610 (9.1)	371 (8.2)	
Fringe counties of metro ≥ 1 million	5,093 (22.8)	10,397 (22.5)	5,972 (24.4) **		541 (21.5)	1,453 (21.7)	1,005 (22.3) **	
Micropolitan counties	2,085 (9.3)	4,427 (9.6)	2,495 (10.2) *		228 (9.1)	630 (9.4)	404 (9.0) *	
Non-metro or micropolitan counties	1,377 (6.2)	2939 (6.4)	1780 (7.3) *		166 (6.6)	407 (6.1)	285 (6.3) *	
Mean household income by zip code (%)				<0.001				<0.001
0-25th percentile	8,305 (37.2)	17,319 (37.4)	7,606 (31.0)		885 (35.2) **	2,374 (35.5) **	1,349 (30.0)	
26th to 50th percentile	5,750 (25.7)	12,180 (26.3)	6,484 (26.4)		619 (24.7)	1,779 (26.6)	1,157 (25.7)	
51st to 75th percentile	4,958 (22.2)	10,076 (21.8)	5,696 (23.2)		588 (23.4)	1,473 (22.0)	1,100 (24.4)	
76th to 100th percentile	3,324 (14.9)	6,706 (14.5)	4,731 (19.3)		419 (16.7) **	1,062 (15.9) **	895 (19.9)	
Primary payer for insurance (%)				<0.001				<0.001
Medicare	3,230 (14.5)	13,397 (28.9)	21,347 (87.1)		481 (19.2) ***	2,263 (33.8) *	3,996 (88.8) **	
Medicaid	8,670 (38.8)	12,635 (27.3)	613 (2.5)		1,010 (40.2)	1,776 (26.6)	96 (2.1)	
Private insurance	4765 (21.3)	12,317 (26.6)	1,985 (8.1)		587 (23.4) *	1,826 (27.3)	329 (7.3)	
Self-pay	1,307 (5.9)	2,392 (5.2)	361 (1.5)		282 (11.2) ***	491 (7.3) ***	26 (0.6) ***	
No pay	416 (1.9)	557 (1.2)	13 (0.1)		36 (1.4)	39 (0.6) ***	3 (0.1)	
Other	3,949 (17.7)	4,983 (10.8)	198 (0.8)		115 (4.6) ***	293 (4.4) ***	51 (1.1) *	

[†]Routine discharge is included in the total percentage and is shown in the main tables

Abbreviations: ICF: Intermediate care facility, SNF: Skilled nursing facility.

[a] p values that are marked represent ANOVA analyses. Cirrhotics without muscle loss vs cirrhotics with muscle loss, between each age group: * p <0.05, ** p <0.01, *** p <0.001.

Supplementary Table S5. Linear regression analysis for hospitalized general medicine and cirrhosis patients predicting variables associated with the Elixhauser comorbidity score.

Age categories as categorical variables and muscle loss phenotype as a continuous independent variable

	Cirrhosis Elixhauser comorbidity score unadjusted (95% CI)	Cirrhosis Elixhauser comorbidity score adjusted (95% CI)	GMP Elixhauser comorbidity score unadjusted (95% CI)	GMP Elixhauser comorbidity score adjusted (95% CI)
Age category 51-65y (compared to ≤50)	2.14 (1.96-2.33)	1.79 (1.61-1.96)	3.16 (3.10-3.22)	2.93 (2.87-2.99)
Age category >65y (compared to <≤50)	6.20 (5.99-6.41)	5.43 (5.24-5.62)	6.41 (6.36-6.46)	5.94 (5.89-5.99)
Muscle loss phenotype (%)	14.49 (14.28-14.70)	14.15 (13.95-14.35)	18.14 (17.98-18.31)	16.52 (16.37-16.68)

Cirrhosis/GMP as categorical variable and age as a continuous independent variable

	Elixhauser comorbidity score unadjusted (95% CI)	Elixhauser comorbidity score adjusted (95% CI)
Cirrhosis (vs GMP)	10.75 (10.69−10.82)	10.57 (10.51−10.64)
Age (y)	0.150 (0.149−0.151)	0.145 (0.144−0.146)

Supplementary Table S6. Multivariate logistic regression of in-hospital mortality in age groups (≤50, 51-65, >65) of hospitalized cirrhotic patients.

Age categories (years)	≤50 years old		51−65 years old		>65 years old	
	Exponentiated coefficient (95% CI)	*p*-value	Exponentiated coefficient (95% CI)	*p* value	Exponentiated coefficient (95% CI)	*p*-value
Gender (Female)	1.00 [1.00, 1.00]	0.63	1.00 [0.99, 1.00]	0.22	1.01 [1.00, 1.01]	0.08
Age	1.00 [1.00, 1.00]	0.07	1.00 [1.00, 1.00]	<0.001	1.00 [1.00, 1.00]	0.29
Muscle loss phenotype	1.09 [1.08, 1.10]	<0.001	1.08 [1.08, 1.09]	<0.001	1.09 [1.08, 1.10]	<0.001
Race (black)						
White	0.98 [0.97, 0.99]	0.01	0.99 [0.98, 1.00]	0.01	0.97 [0.96, 0.98]	<0.001
Hispanic	0.98 [0.97, 1.00]	0.05	0.98 [0.97, 0.99]	0.001	0.99 [0.97, 1.00]	0.18
Other	1.00 [0.98, 1.02]	0.62	0.99 [0.98, 1.01]	0.80	1.00 [0.98, 1.02]	0.73
Comorbidities						
Acute kidney injury	1.04 [1.03, 1.05]	<0.001	1.03 [1.02, 1.04]	<0.001	1.02 [1.01, 1.03]	<0.001
Alcohol abuse	0.99 [0.99, 1.00]	0.54	0.99 [0.98, 0.99]	<0.001	1.00 [0.99, 1.00]	0.82
Anemia	0.99 [0.98, 1.01]	0.66	0.99 [0.98, 1.00]	0.49	0.98 [0.96, 1.00]	0.06
Chronic lung disease	0.99 [0.98, 1.00]	0.29	0.99 [0.99, 1.00]	0.79	1.00 [0.99, 1.01]	0.18
Coagulopathy	1.05 [1.05, 1.06]	<0.001	1.06 [1.06, 1.07]	<0.001	1.04 [1.03, 1.05]	<0.001
Congestive heart failure	1.04 [1.02, 1.05]	<0.001	1.02 [1.02, 1.03]	<0.001	1.02 [1.01, 1.03]	<0.001
Diabetes (uncomplicated)	0.97 [0.96, 0.97]	<0.001	0.96 [0.96, 0.97]	<0.001	0.97 [0.96, 0.98]	<0.001
Diabetes (complicated)	0.98 [0.97, 1.00]	0.15	0.95 [0.94, 0.96]	<0.001	0.96 [0.95, 0.97]	0.01
Metastatic cancer	1.18 [1.13, 1.23]	<0.001	1.13 [1.11, 1.16]	<0.001	1.09 [1.07, 1.11]	<0.001
Solid tumors	1.02 [1.00, 1.05]	0.02	1.02 [1.01, 1.03]	<0.001	1.03 [1.01, 1.04]	<0.001

Multiple R square (≤50): 0.14; $p < 2.2e-16$, Multiple R square (51−65): 0.12; $p < 2.2e-16$, Multiple R square (>65): 0.09; $p < 2.2e-16$. Abbreviations: CI: Confidence interval

Supplementary Table S7. Multivariate linear regression analysis predicting variables associated with increased length of stay in age groups (≤50, 51-65, >65) of hospitalized cirrhotic patients.

Age categories (years)	≤50 y		51−65 y		>65 y	
	Exponentiated coefficient (95% CI)	p-value	Exponentiated coefficient (95% CI)	p-value	Exponentiated coefficient (95% CI)	p-value
Gender (Female)	0.95 [0.93, 0.97]	<0.001	0.97 [0.96, 0.98]	<0.001	0.97 [0.96, 0.99]	0.002
Age	0.99 [0.99, 0.99]	<0.001	1.00 [1.00, 1.00]	<0.001	1.00 [0.99, 1.00]	0.08
Muscle loss phenotype	1.97 [1.91, 2.03]	<0.001	1.79 [1.76, 1.83]	<0.001	1.51 [1.48, 1.55]	<0.001
Race (black)						
White	0.95 [0.94, 1.00]	0.09	0.97 [0.96, 1.00]	0.29	0.87 [0.85, 0.90]	<0.001
Hispanic	0.95 [0.91, 0.98]	0.004	0.95 [0.92, 0.97]	<0.001	0.88 [0.85, 0.91]	<0.001
Other	1.00 [0.96, 1.05]	0.76	1.03 [0.99, 1.06]	0.10	0.95 [0.91, 0.99]	0.02
Comorbidities						
Acute kidney injury	1.29 [1.25, 1.33]	<0.001	1.18 [1.17, 1.20]	<0.001	1.11 [1.09, 1.13]	<0.001
Alcohol abuse	0.96 [0.94, 0.97]	<0.001	0.96 [0.94, 0.97]	<0.001	0.96 [0.95, 0.98]	<0.001
Anemia	1.09 [1.04, 1.13]	<0.001	1.02 [0.99, 1.05]	0.18	1.04 [0.99, 1.07]	0.05
Chronic lung disease	1.04 [1.01, 1.07]	0.001	1.07 [1.05, 1.08]	0.001	1.09 [1.07, 1.11]	0.001
Coagulopathy	1.26 [1.23, 1.29]	<0.001	1.24 [1.23, 1.26]	<0.001	1.17 [1.15, 1.19]	<0.001
Congestive heart failure	1.24 [1.20, 1.29]	<0.001	1.19 [1.17, 1.22]	<0.001	1.12 [1.10, 1.14]	<0.001
Diabetes (uncomplicated)	0.99 [0.97, 1.02]	0.45	0.96 [0.94, 0.97]	<0.001	0.94 [0.93, 0.96]	<0.001
Diabetes (complicated)	1.04 [0.99, 1.09]	0.07	1.07 [1.05 1.10]	<0.001	1.02 [1.00 1.05]	0.04
Metastatic cancer	1.06 [0.95, 1.20]	0.30	1.14 [1.09, 1.20]	<0.001	1.07 [1.02, 1.12]	0.004
Solid tumors	1.12 [1.05, 1.20]	<0.001	1.08 [1.05, 1.12]	<0.001	1.03 [0.99, 1.06]	0.12

Multiple R square (≤50): 0.13; $p < 2.2e\text{-}16$, Multiple R square (51−65): 0.12; $p < 2.2e\text{-}16$, Multiple R square (>65): 0.08; $p < 2.2e\text{-}16$. Abbreviations: CI: Confidence interval

Supplementary Table S8. Multivariate linear regression predicting variables associated with log transformed cost of hospitalization in age groups (≤50, 51−65, >65) of cirrhotics patients.

Age categories (years)	≤50 y		51-65 y		>65 y	
	Exponentiated coefficient (95% CI)	p-value	Exponentiated coefficient (95% CI)	p-value	Exponentiated coefficient (95% CI)	p-value
Gender (Female)	0.96 [0.94, 0.99]	0.01	1.00 [0.99, 1.02]	0.74	1.01 [0.97, 1.04]	0.08
Age	0.99 [0.98, 0.99]	<0.001	1.00 [1.00, 1.00]	<0.001	0.99 [0.99, 0.99]	<0.001
Muscle loss phenotype	2.34 [2.25, 2.43]	<0.001	2.07 [2.02, 2.12]	<0.001	1.67 [1.63, 1.72]	<0.001
Race (black)						
White	0.99 [0.95, 1.04]	0.90	1.02 [0.99, 1.05]	0.07	0.88 [0.84, 0.91]	<0.001
Hispanic	1.04 [0.99, 1.09]	0.05	1.01 [0.97, 1.04]	0.45	0.92 [0.88, 0.97]	0.002
Other	1.13 [1.06, 1.20]	<0.001	1.17 [1.12, 1.22]	<0.001	1.12 [1.05, 1.18]	<0.001
Comorbidities						
Acute kidney injury	1.39 [1.34, 1.44]	<0.001	1.23 [1.21, 1.26]	<0.001	1.13 [1.10, 1.15]	<0.001
Alcohol abuse	0.90 [0.88, 0.92]	<0.001	0.89 [0.88, 0.91]	<0.001	0.94 [0.91, 0.96]	<0.001
Anemia	1.23 [1.17, 1.30]	<0.001	1.11 [1.07, 1.16]	<0.001	1.11 [1.06, 1.16]	<0.001
Chronic lung disease	1.02 [0.98, 1.05]	0.21	1.07 [1.05, 1.09]	<0.001	1.13 [1.11, 1.16]	<0.001
Coagulopathy	1.45 [1.42, 1.49]	<0.001	1.42 [1.39, 1.44]	<0.001	1.32 [1.30, 1.35]	<0.001
Congestive heart failure	1.27 [1.21, 1.34]	<0.001	1.18 [1.15, 1.22]	<0.001	1.13 [1.10, 1.16]	0.006
Diabetes (uncomplicated)	1.01 [0.97, 1.04]	0.62	0.96 [0.94, 0.98]	<0.001	0.93 [0.91, 0.95]	<0.001
Diabetes (complicated)	1.06 [1.00, 1.13]	0.03	1.09 [1.05, 1.12]	<0.001	1.04 [1.00, 1.07]	0.01
Metastatic cancer	1.29 [1.11, 1.50]	<0.001	1.23 [1.15, 1.31]	<0.001	1.22 [1.15, 1.30]	<0.001
Solid tumors	1.37 [1.25, 1.50]	<0.001	1.36 [1.31, 1.42]	<0.001	1.14 [1.09, 1.19]	<0.001

Multiple R square (≤50): 0.14; p < 2.2e-16, Multiple R square (51-65): 0.12; *p* < 2.2e-16, Multiple R square (>65): 0.09; *p* < 2.2e-16. Abbreviations: CI: Confidence interval.
